# Altair-LSFM A High-Resolution, Easy-to-Build Light-Sheet Microscope for Sub-Cellular Imaging

**DOI:** 10.1101/2025.04.08.647739

**Published:** 2025-11-07

**Authors:** John Haug, Seweryn Gałecki, Hsin-Yu Lin, Xiaoding Wang, Kevin M. Dean

**Affiliations:** 1 Lyda Hill Department of Bioinformatics, UT Southwestern Medical Center, Dallas, TX 75390, USA.; 2 Cecil H. and Ida Green Center for Systems Biology, UT Southwestern Medical Center, Dallas, TX 75390, USA.; 3 Department of Systems Biology and Engineering, Silesian University of Technology, Akademicka 16, 44-100 Gliwice, Poland.

## Abstract

Although several open-source, easy-to-assemble light-sheet microscope platforms already exist—such as mesoSPIM, OpenSPIM, and OpenSpin—they are optimized for imaging large specimens and lack the resolution required to visualize sub-cellular features, such as organelles or cytoskeletal architectures. In contrast, Latice Light-Sheet Microscopy (LLSM) achieves the resolution necessary to resolve such fine structures but, in its open-source implementation, can be alignment- and maintenance-intensive, often requiring specialist expertise. To address this gap, we developed Altair-LSFM, a high-resolution, open-source, sample-scanning light-sheet microscope specifically designed for sub-cellular imaging. By optimizing the optical pathway in silico, we created a custom baseplate that greatly simplifies alignment and assembly. The system integrates streamlined optoelectronics and optomechanics with seamless operation through our open-source software, *navigate*. Altair-LSFM achieves lateral and axial resolutions of approximately 235 nm and 350 nm, respectively, across a 266-micron field of view after deconvolution. We validate the system’s capabilities by imaging sub-diffraction fluorescent nanospheres and visualizing fine structural details in mammalian cells, including microtubules, actin filaments, nuclei, and Golgi apparatus. We further demonstrate its live-cell imaging capabilities by visualizing microtubules and vimentin intermediate filaments in actively migrating cells.

## Introduction

Light-sheet fluorescence microscopy (LSFM) has revolutionized volumetric imaging by enabling rapid, minimally invasive 3D investigations of diverse biological specimens^1^. By illuminating the sample with a thin sheet of light from the side and capturing 2D images in a highly parallel format, LSFM dramatically reduces photobleaching and out-of-focus blur. Although the optical foundations of LSFM were established as early as 1903 and later adapted for cleared specimens^2^, it was the demonstration of LSFM on living, developing embryos in 2004^1^ that triggered a wave of innovation, leading to specialized variants tailored to diverse biological contexts. Collectively, these methods have facilitated the long-term tracking of cells through embryological development^3^, the mapping of brain architecture^4^ and activation patterns^5^, and much more, making LSFM indispensable for a wide array of dynamic, 3D imaging applications.

While these milestones underscore LSFM’s transformative potential, it wasn’t until the early 2010s that researchers harnessed LSFM for sub-cellular imaging^6^. Achieving this level of detail requires optimization of both resolution and sensitivity, parameters fundamentally governed by the numerical aperture (NA) of the microscope objectives used. In LSFM, lateral resolution depends on the fluorophore’s emission wavelength and the NA of the detection objective, whereas photon collection efficiency scales with the square of the detection NA. Consequently, high-NA objectives are essential for resolving fine, low abundance biological structures while maximizing signal collection. Axial resolution, in turn, is set by the detection objective’s depth of focus and the thickness of the illumination beam. When the illumination beam is thinner than the depth of focus, its thickness defines the axial resolution^7^; conversely, when the beam is thicker than the depth of focus, the depth of focus defines the axial resolution, and fluorescence elicited outside this region contributes to the image as blur. Importantly, there is a trade-off between field of view, axial resolution, and NA: pushing for high axial resolution often constrains the accessible field of view, necessitating careful mechanical and optical design choices.

Bounded by these fundamental constraints, several methods have been developed to achieve sub-cellular resolution in LSFM. For example, one can illuminate the specimen with a propagation-invariant beam^6^ or an optical lattice^8^. This can be done coherently, as in Lattice Light-Sheet Microscopy (LLSM)^8^, or incoherently, as in Field Synthesis^9^. However, the four-beam “square” optical lattice, which was used in 16 of the 20 figure subpanels in the original LLSM study, was later found to provide little measurable improvement in resolution or sectioning compared to a traditional Gaussian beam^10^. Another approach, dual view inverted Selective Plane Illumination Microscopy (diSPIM), captures images from multiple orthogonal perspectives and computationally fuses them using iterative deconvolution, significantly improving axial resolution^11^. However, this method requires precise image registration and intensive computational processing. Axially Swept Light-sheet Microscopy (ASLM)^12^ extends the field of view while maintaining high axial resolution but operates at lower speeds and sensitivity compared to LLSM and diSPIM, making it less suitable for fast volumetric imaging. Oblique plane microscopy (OPM) offers another alternative by imaging an obliquely launched light-sheet with a non-coaxial and complex optical train, allowing for single-objective light-sheet imaging but introducing substantial alignment challenges^13^. While these techniques offer powerful solutions for sub-cellular imaging, they all require expert assembly and routine alignment, limiting their widespread adoption. Turn-key commercial variants, such as the ZEISS Lattice Lightsheet 7, offer automated operation and high stability but remain costly and allow limited end-user modifiability. As a result, there remains a critical need for a high-resolution, accessible LSFM system that combines state-of-the-art imaging performance with straightforward assembly, reproducibility, and lower cost.

To address these limitations, we developed Altair-LSFM, a high-resolution, open-source light-sheet microscope that achieves sub-cellular detail while remaining accessible and easy to use. Altair-LSFM is built upon two guiding optical principles. First, in LLSM, the sole improvement in lateral resolution comes from the use of a higher-NA detection objective, which we incorporate to maximize both resolution and photon collection efficiency. Second, when diffraction effects are fully accounted for, a tightly focused Gaussian beam achieves a beam waist and propagation length that is comparable to that of a square lattice, eliminating the need for specialized optical components while preserving high axial resolution. By leveraging these principles, Altair-LSFM delivers optical performance on par with LLSM but without the added design complexity of LLSM. To streamline assembly and ensure reproducibility, we designed the optical layout for Altair-LSFM in silico, enabling a pre-defined optical alignment with minimal degrees of freedom. A custom-machined baseplate with precisely positioned dowel pins locks optical components into place, minimizing degrees of freedom and removing the need for fine manual adjustments. Additionally, by simplifying the optomechanical design and integrating compact optoelectronics, Altair-LSFM reduces system complexity, making advanced light-sheet imaging more practical for a wider range of laboratories.

By combining high-resolution imaging with an accessible and reproducible design, Altair-LSFM addresses a critical gap in LSFM—bringing sub-cellular imaging capabilities to a broader scientific community. Its reliance on fundamental microscopy principles rather than overly complex optical systems ensures both performance and simplicity, while its modular architecture allows for straightforward assembly and operation. By eliminating the need for specialized optics and intricate alignment procedures, Altair-LSFM significantly lowers the barrier to adoption, making advanced light-sheet imaging feasible for laboratories that lack the resources or expertise to implement more complex systems. This combination of performance, accessibility, and scalability establishes Altair-LSFM as a powerful and practical solution for a wide range of laboratories.

## Results

### Survey of Open-Source LSFM Designs.

Before designing Altair-LSFM, we first evaluated existing open-source LSFM implementations to identify common design features, constraints, and trade-offs (Table 1). Many systems, such as UC2^14^, pLSM^15^, and EduSPIM^16^, were explicitly developed with cost-effectiveness in mind, relying on low-cost components and simplified designs to maximize accessibility. Others, including OpenSPIM^17,18^, OpenSPIN^19^, and mesoSPIM^20,21^, were optimized for imaging large specimens, such as developing embryos or chemically cleared tissues. Most of these systems employed modular construction methods based on rail carriers or cage systems, which, while reducing alignment complexity compared to free-space optics, still retain degrees of freedom that can lead to misalignment and increase setup difficulty. Moreover, these microscopes generally operate at low magnification and low NA, limiting their ability to resolve sub-cellular structures. Among the surveyed designs, only diSPIM^11,22^ was explicitly developed for sub-cellular imaging, built with a dovetail-based system for precise optical alignment. However, diSPIM’s most widely deployed configuration uses NA 0.8 objectives, which limits its photon collection efficiency and resolution. Consequently, the cell biology community lacks an open-source light-sheet microscope that combines state-of-the-art resolution with ease of assembly, robust optical alignment, and streamlined computational processing.

### Design Principles of Altair-LSFM.

Building on these findings, we designed Altair-LSFM to achieve performance comparable to the open-source variant of LLSM^8^ while maintaining a compact footprint and streamlined assembly. Although cost-effectiveness was an important consideration throughout the design process, achieving sensitive, high-resolution imaging necessitated the use of high-NA optics, precision stages, stable laser sources, and high-performance, low-noise, high quantum efficiency cameras, all of which inherently increase system cost. To streamline procurement and integration, we minimized the number of required manufacturers while maintaining high-performance components. Including our optical table and laser source, the estimated price for Altair-LSFM is $150,000. A detailed list of all system components, their sources, and associated costs is provided in Supplementary Tables 1 and 2, and a broader discussion of the design trade-offs, including the rationale for proprietary versus open-source hardware, and associated cost-benefit considerations, is provided in Supplementary Note 1.

This initial iteration of Altair-LSFM is specifically designed for imaging thin, adherent cells on 5 mm glass coverslips in aqueous media (n ~ 1.33). For imaging such specimens, a sample-scanning approach is preferred over a light-sheet-scanning approach, as it minimizes the optical path length through the specimen, enabling use of more tightly focused illumination beams that improve axial resolution (Supplementary Figure 1). If optical tiling is employed, Altair-ASLM could also be used for imaging expansion microscopy specimens^4^. While Altair-LSFM could be used for superficial imaging in semi-transparent embryos, systems implementing multiview illumination and detection schemes are generally better suited for such specimens^23^. Similarly, cleared tissue imaging typically requires high-refractive index media (~1.45–1.56) and solvent-compatible objectives, along with methods such as ASLM or diSPIM that decouple the tradeoff between field of view and axial resolution^24,25^.

**Figure 1.**
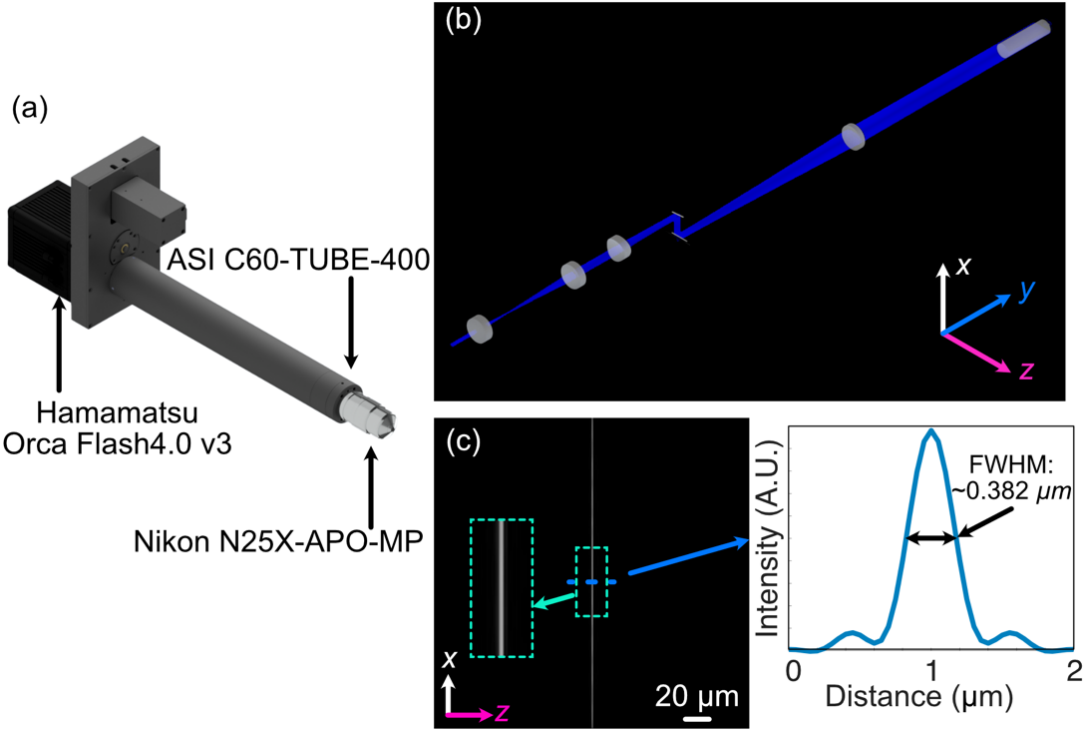
(a) Rendering of the detection arm elements. (b) Zemax Opticstudio layout and beam path of optimized illumination arm, where L1, L2, L3, and L4 are 30-, 80-, 75-, and 250-mm achromatic doublets, respectively, and ILO is the TL20X-MPL illumination objective. (c) The simulated light-sheet beam profile in the xz plane at the focus of the illumination objective. The inset shows an enlarged region of the illumination light-sheet, highlighting light-sheet thickness and uniformity. (d) The cross-sectional profile through the center of the light-sheet beam profile in (c), where the FWHM of the light-sheet was found to be 0.382 μm.

Altair-LSFM is configured with a detection path that is nearly identical to that of LLSM, ensuring similar resolution (~230 nm × 230 nm × 370 nm) and photon collection efficiency. Specifically, it includes a 25x NA 1.1 water-dipping physiology objective (Nikon N25X-APO-MP) with a 400 mm achromatic tube lens (Applied Scientific Instrumentation), ensuring Nyquist sampling (130 nm pixel size) across the full width of a standard 25 mm CMOS camera (Hamamatsu Orca Flash 4.0 v3), yielding a total field of view of 266 microns. Emission filters were positioned in the focusing space immediately before the camera, and the entire detection assembly was built around a dovetail-based tube system, to ensure robust alignment and mechanical stability (Figure 1a). To facilitate precise axial positioning and accommodate different sample types, the entire detection assembly was mounted on a 50 mm travel focusing stage.

With the detection path establishing the necessary criteria for resolution, field of view, and optical alignment (e.g., beam height), we next designed the illumination system. In LSFM, the foci of the illumination and detection objectives must precisely overlap without mechanical interference, limiting the choice of compatible objectives. To meet these requirements, we selected a 20x NA 0.6 long-working distance water immersion objective (Thorlabs TL20X-MPL). The spacing between this combination of illumination and detection objectives limits the size of usable coverslips to 5 mm, a constraint that is shared by the original LLSM design. While handling and mounting small coverslips can be challenging, we addressed this by designing a custom-machined coverslip holder to streamline the mounting process. For users wishing to accommodate larger coverslips, the Nikon 25x objective can be substituted with a Zeiss W Plan-Apochromat 20x/1.0, whose slimmer form factor allows the co-focus between the illumination and detection objectives to occur beyond the physical body of the lenses, enabling the use of virtually any coverslip size^26^. Guided by these constraints, we selected optical components capable of generating a theoretically diffraction-limited light-sheet using straightforward magnification calculations.

The complete illumination system was designed for a collimated input beam with a 2 mm diameter, which first passes through an achromatic doublet lens (f = 30 mm) and then a second achromatic doublet (f = 80 mm) that expands and re-collimates it. After expansion, the beam passes through a rectangular aperture before reaching an achromatic cylindrical lens (f = 75 mm). The rectangular aperture is conjugate to the back pupil plane of the cylindrical lens, enabling precise tuning of the light-sheet’s NA, and consequently its thickness and propagation length. This adjustability allows optimization of the light-sheet for specimens of different thicknesses. The cylindrical lens focuses the beam in one direction to form the initial light-sheet, and its focal length was chosen to provide sufficient spacing between optical elements for practical assembly. The shaped beam is then directed onto a resonant galvanometer, which improves illumination uniformity by rapidly pivoting the light-sheet to average out shadowing artifacts arising from scattering and absorption within the sample (conceptual example shown in Supplementary Figure 2)^27,28^. After reflection from a 45-degree tilted mirror, the beam is relayed through an achromatic doublet (f = 250 mm) before entering the back aperture of the illumination objective, where it is finally focused onto the sample. This optical arrangement ensures a well-defined, dynamically pivoted light-sheet that provides uniform illumination while mitigating shadowing effects.

### In Silico Optimization of Altair-LSFM.

To ensure optimal illumination performance, we modeled the full illumination pathway of Altair-LSFM in Zemax OpticStudio (Ansys), systematically optimizing the relative placement of every optical element to achieve the desired focusing and collimation properties (Figure 1b). Each lens was iteratively adjusted to minimize aberrations and ensure precise beam shaping, enabling the formation of a well-defined light-sheet. The design was centered around a 488 nm illumination wavelength, with spatial axes defined following standard conventions: the Y-dimension represents the laser propagation direction, Z corresponds to the detection axis, and X is orthogonal to both. The final illumination system, depicted in Figure 1b, was optimized to generate a diffraction-limited light-sheet with a full-width-half-maximum (FWHM) of ~0.385 μm in Z, spanning the full 266 μm field of view, as shown in Figures 1c-d.

Beyond idealized modeling, designing a physically realizable system requires an understanding of how fabrication tolerances affect optical performance. To assess system robustness, we performed a tolerance analysis, which quantifies sensitivity to mechanical perturbations. This analysis evaluates how small positional or angular deviations of optical elements—caused by manufacturing imperfections—impact key performance metrics such as light-sheet thickness and displacement from the ideal position, allowing us to systematically evaluate system stability (Supplementary Figure 3a). The perturbations analyzed were based on standard machining tolerances, typically ±0.005 inches, with higher-precision machining achievable at ±0.002 inches at increased cost. Given that Altair-LSFM was designed assuming the use of Polaris mounts, which incorporate DIN-7m6 ground dowel pins to aid with alignment, we considered the impact of angular misalignments caused by dowel pin positioning errors. In the worst-case scenario—where one dowel pin was offset by +0.005 inches and the other by −0.005 inches—the resulting angular deviation was ~1.45 degrees (Supplementary Figure 3b). To further assess system resilience, we conducted Monte Carlo simulations incorporating these perturbations, simulating a range of misalignment scenarios to quantify their effect on light-sheet performance. Our results showed that finer machining tolerances resulted in a worst-case performance closer to the nominal system, as visualized in Supplementary Figure 3c, which compares the nominal, best, and worst configurations. Notably, the analysis identified that angular offsets in the galvo mirror had the most significant impact on light-sheet quality, highlighting the importance of tighter machining tolerances for this component to maximize system stability and performance.

### Optomechanical Design of Altair-LSFM.

Based on our simulation results, standard machining tolerances were deemed sufficient to construct a custom baseplate that ensures robust alignment and a compact, plug-and-play design. Unlike cage- or rail-based systems, custom baseplates minimize variability by enforcing a fixed spatial relationship between optical components, enabling assembly by non-experts. Where possible, we eliminated all unnecessary degrees of freedom, restricting manual adjustments to only a few critical components. Specifically, we retained laser collimation (tip/tilt/axial position), galvo rotation, folding mirror alignment (tip/tilt), and objective positioning (tip/tilt/axial position).

**Figure 2.**
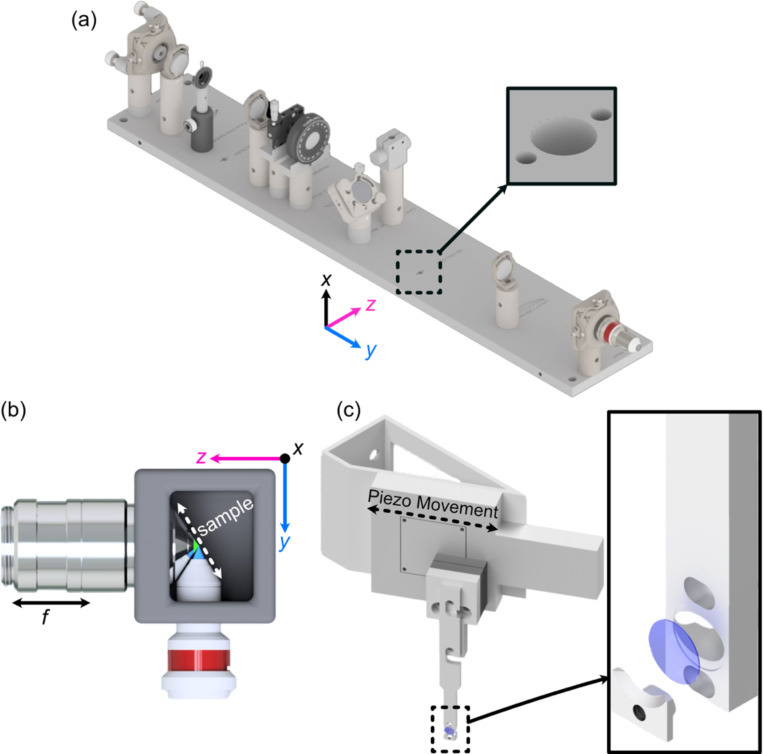
(a) Rendering of the completed illumination arm baseplate, with an inset showing the dowel pin holes compatible with the Polaris mounting line from Thorlabs. (b) Overhead view of the imaging configuration of our system, where our detection objective and illumination objectives are placed orthogonal to each other and the sample is scanned diagonally in the space between them in the axial direction shown by the white dashed line. (c) Rendering of our sample mounting and translation system. Here, a piezo motor is mounted onto an angled adapter to allow precise translation over the diagonal region between the objectives. Our custom 5 mm coverslip sample holder is also featured, where the inset shows an exploded assembly of the holder.

To translate the numerically optimized positions of each optical element into a manufacturable design, the coordinates were imported into computer-aided design (CAD) software (Autodesk Inventor), ensuring precise positioning of all associated optomechanics. Where possible, Polaris optical posts and mounts were used to maintain consistency in mounting schemes and element heights. For components where a commercially available Polaris-compatible mount did not exist, such as the rotation mount (Thorlabs RSP1) for the cylindrical lens and the horizontal aperture (Thorlabs VA100), custom adapters were developed to seamlessly integrate them into the system. This approach allowed us to account for the offset between the optical element and its mechanical mount, ensuring that the baseplate was precisely machined with dowel pin locations and mounting holes (Figure 2a, Supplementary Figure 4). Additionally, the baseplate features four mounting holes at its corners, spaced such that it can be directly secured to an optical table or elevated using additional posts, allowing for easy adjustment of the illumination path height. This modular, precision-engineered design is meant to ensure both ease of use and general mechanical stability, enabling integration of Altair-LSFM into alternative experimental setups.

Beyond the illumination path, the full microscope system is visualized in Supplementary Figure 5. It incorporates a variety of translational and custom mounting elements to facilitate precise sample positioning and stable imaging. A sample chamber was designed and 3D printed to match the working distances and clearances of the chosen illumination and detection objectives, offering two port configurations: one for traditional orthogonal imaging and another linear configuration that allows direct imaging of the light-sheet itself (Figure 2b, Supplementary Figure 6). Each port is equipped with two sets of O-rings, creating a liquid-proof seal around the objectives while still permitting smooth translation of the detection objective for focusing. Additionally, the snug fit of the O-rings naturally guide the user toward proper positioning of the illumination and detection objectives, decreasing the likelihood of alignment errors. The sample positioning assembly consists of three motorized translation stages (Applied Scientific Instrumentation), enabling precise positioning of the sample in x, y, and z. To enable rapid Z-stack acquisition, we designed an angled bracket (θ = 29.5 degrees) for mounting a high-speed piezo (PiezoConcept HS1.100), which attaches directly to the sample positioning stages (Figure 2c). The sample is secured using a custom-designed sample holder for 5 mm glass coverslips, which features a clam-based mechanism-the coverslip is placed within a circular recess and secured in place by a screw-down clamp. Due to the angled sample scanning configuration, our collected image stacks must undergo a deskewing operation. All custom component designs, and deskewing software, are available for download at https://thedeanlab.github.io/altair.

### Optoelectronic Design of Altair-LSFM.

In addition to simplifying the optomechanical design, we also streamlined the electronics and control architecture of Altair-LSFM to minimize complexity and improve system integration. To achieve this, we consolidated all control electronics into a single controller (TG16-BASIC, Applied Scientific Instrumentation), which manages the operation of all linear translation stages (X, Y, Z, and F), as well as the power supply for the resonant galvo and sample scanning piezo. This approach significantly reduces the number of auxiliary controllers and power supplies, simplifying the physical setup. Currently, all timing operations are performed using a 32-channel analog output device (PCIe-6738, National Instruments), which is responsible for generating the global trigger, controlling the camera’s external trigger, modulating the laser through analog and digital signals, setting the piezo control voltage, and providing the DC voltage for adjusting the resonant galvo amplitude. The resonant galvo used for shadow reduction operates at 4 kHz, ensuring that it is not rate-limiting for any acquisition mode described here. An overview of the electronics used in the system, along with an associated wiring diagram, is provided in Supplementary Figure 7 and Supplementary Table 3.

All control electronics are operated through *navigate*, our open-source light-sheet microscope control software, which integrates hardware coordination, waveform generation, and data acquisition within a unified software environment^29^. The combined performance of the control electronics and *navigate* defines the system’s maximum temporal resolution. Mechanically, the acquisition of a z-stack is constrained by the response time of the sample-scanning piezo. Approximating the piezo as a first-order system gives a characteristic response time of ~0.35/f (seconds), where f is the actuator’s resonant frequency^30^. For our piezo (HS100, PiezoConcept), this gives an ideal response time of ~0.23 ms; however, accounting for additional physical considerations such as the weight of a sample holder, we expect this value to realistically be on the order of 1–5 ms for small step sizes. We also evaluated the rate at which a Z-stack could be acquired with *navigate* using representative settings for coverslip-mounted cells (50-micron z-stack, 0.25-micron step size, camera field of view of 512×2048). With a 10 ms exposure time, the system achieved image acquisition rates as fast as 62.5 Hz, with an average dead time of ~7.25 ms due to camera readout and piezo stepping (Supplementary Table 4). Moreover, as demonstrated previously, the data-writing performance of *navigate* varies slightly depending on imaging parameters (e.g., number of z-slices and time points, owing to metadata overhead), with write speeds surpassing 1 gigavoxel/s under optimal conditions^29^. Consequently, the integrated hardware and control software establish a unified, optoelectronic platform that balances performance, stability, and accessibility for advanced light-sheet applications.

### Alignment and Characterization of Altair-LSFM.

**Figure 3.**
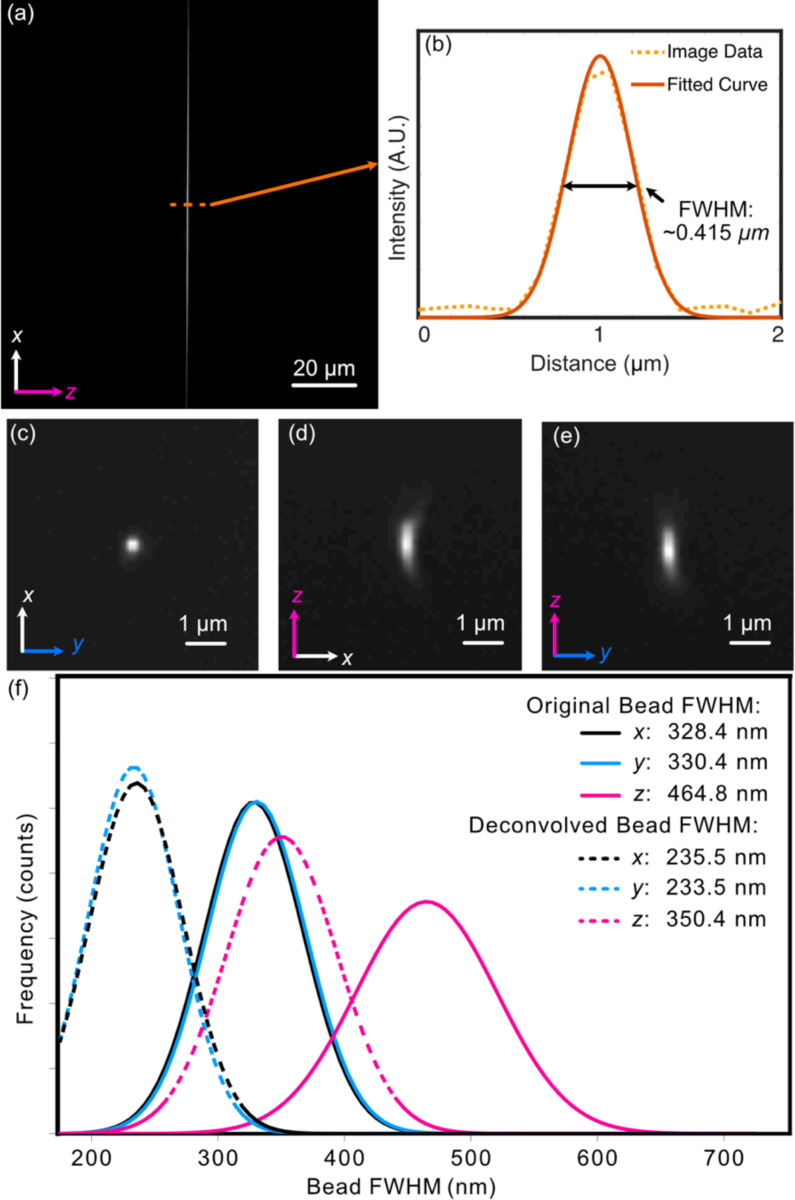
(a) Experimental light-sheet beam profile at the focus. The light-sheet was visualized in a transmission geometry with fluorescence derived from a fluorescein solution. (b) The center cross-section profile of (a), showing both raw data and a fitted curve with a FWHM of ~0.415 μm. (c–e) Maximum-intensity projections for an isolated 100 nm fluorescent bead. All three orthogonal perspectives are provided to reveal any potential optical aberrations. A slight degree of coma is observable in the XZ view. (f) Gaussian-fitted distribution of the FWHM of beads imaged in a z-stack in each dimension both before (solid) and after (dashed) deconvolution.

To evaluate optical performance, we first assembled and aligned the Altair-LSFM illumination system. The fiber-coupled laser source (Oxxius L4CC), which provides four excitation wavelengths (405 nm, 488 nm, 561 nm, and 638 nm), was introduced and collimated using tip/tilt mounts. The collimated beam was then directed onto the resonant galvo, which was rotated to reflect the beam downward toward the optical table. From there, the folding mirror was adjusted to guide the beam along the optical axis of the remaining components. Finally, the lateral position of the illumination objective was fine-tuned to ensure coaxial back-reflections, completing the alignment process. With the optical path aligned, we proceeded to validate system performance by characterizing the generated light-sheet, where visualization of the light-sheet is accomplished by a solution of fluorescein in transmission. As shown in Figure 3a, the light-sheet focus spans the full 266 μm field of view, closely matching our simulation results. Cross-sectional analysis of the full-width-at-half-maximum (FWHM) in the z-dimension, presented in Figure 3b, reveals a z-FWHM of ~0.415 μm.

To assess the system’s resolution, we imaged 100 nm fluorescent beads. Our image-processing pipeline involves first deskewing our acquired volumetric image stack using custom Python routines, and then deconvolution via PetaKit5D^31^ (see Methods). The need for shearing arises when the scan axis does not align with the optical detection axis, as is the case for LLSM and Altair-LSFM when operating in a sample-scanning format, as well as both sample-scanning and laser-scanning OPMs. The point spread function of a single isolated fluorescent bead is shown in Figures 3c-e. The Gaussian-fitted distribution of FWHM measurements, performed on a population of fluorescent beads across a z-stack, is shown in Figure 3f. Prior to deconvolution, the average FWHM values measured across the bead population were 328 nm in x, 330 nm in y, and 464 nm in z. After deconvolution with PetaKit5D, these values improved to 235.5 nm in x, 233.5 nm in y, and 350.4 nm in z, achieving our desired resolution goals for sub-cellular imaging.

### Sub-Cellular Imaging with Altair-LSFM

**Figure 4.**
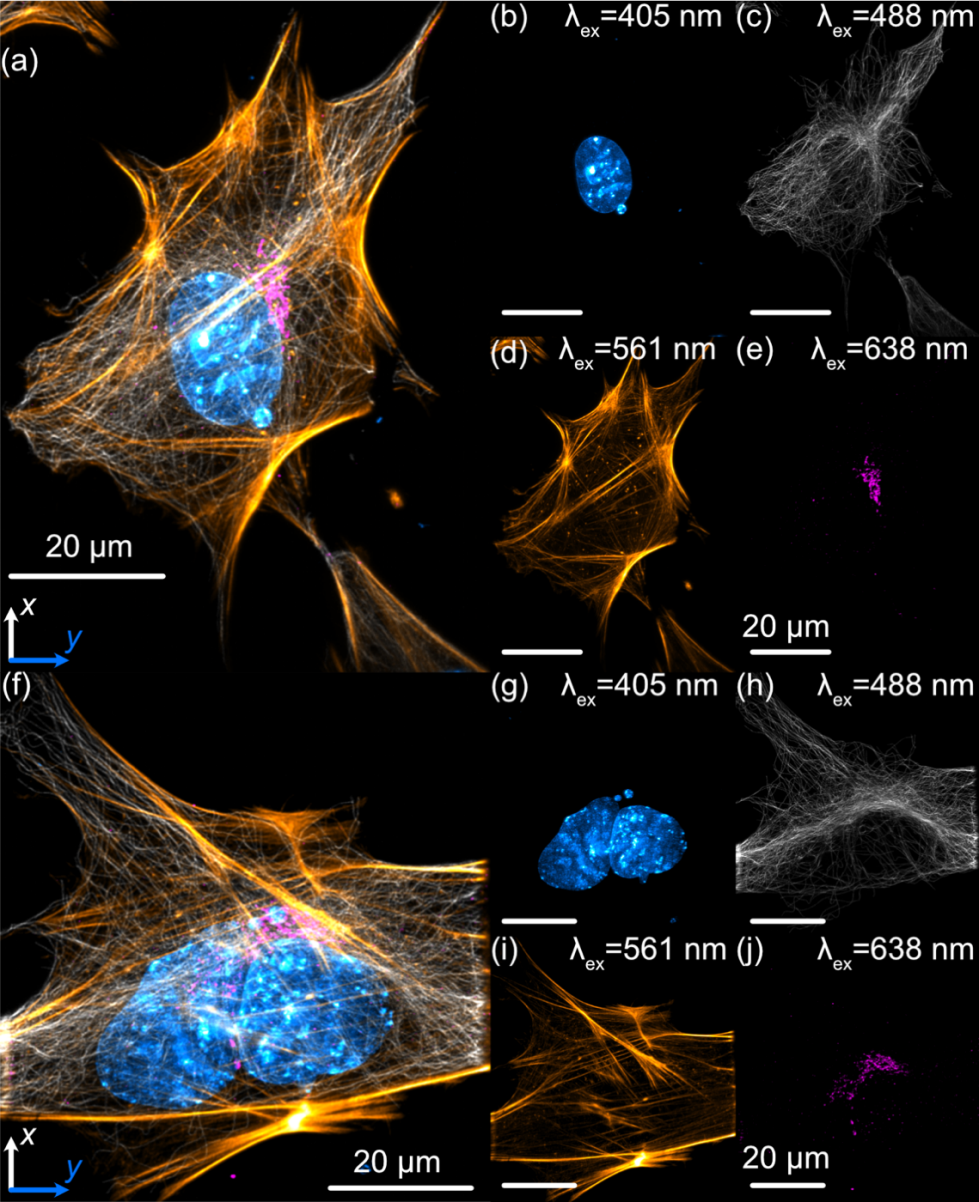
Lateral maximum intensity projections of mouse embryonic fibroblasts (MEFs) fluorescently labeled with nuclei (cyan), tubulin (gray), actin (gold), and the Golgi apparatus (magenta). (a) Maximum intensity projection of the full z-stack in the xy plane. (b–e) Individual channels corresponding to (a): (b) nuclei, (c) microtubules, (d) actin, and (e) Golgi apparatus. (f) Maximum intensity projection of a second z-stack in the xy plane. (g–j) Individual channels corresponding to (f): (g) nuclei, (h) microtubules, (i) actin, and (j) Golgi apparatus. Nuclei were labeled with DAPI, actin filaments with phalloidin, and both microtubules and the Golgi apparatus were stained using indirect immunofluorescence.

**Figure 5.**
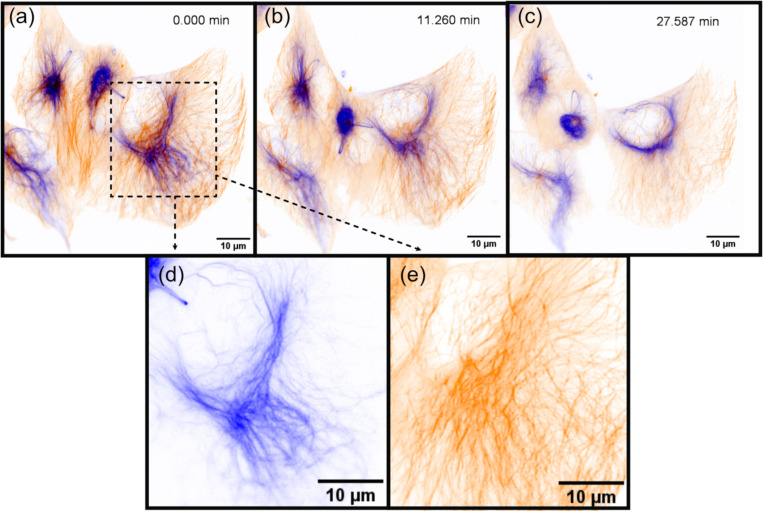
Selected time points from a time-lapse sequence of actively migrating retinal pigment epithelial (RPE) cells, showing vimentin (blue) and microtubules (orange). The series comprises 50 frames; representative time points are displayed.

To demonstrate the imaging capabilities of Altair-LSFM in a biological context, we prepared and imaged mouse embryonic fibroblasts (MEFs) cells stained for multiple subcellular structures. The staining protocol enabled visualization of the nucleus (DAPI, 405 nm, cyan), microtubules (488 nm, gray), actin filaments (561 nm, gold), and the Golgi apparatus (638 nm, magenta), corresponding to the excitation channels of our system. Deconvolved maximum intensity projections of the labeled cells is shown in Figure 4a and Figure 4f, with each individual corresponding fluorescence channel presented in Figures 4b-e and Figures 4g-j. The imaging results reveal fine nucleolar features within the nucleus, with perinuclear Golgi structures distinctly visible. Additionally, stress fibers are clearly resolved in the actin channel, and individual microtubules appear well-defined, highlighting the system’s ability to capture cytoskeletal structures with high resolution. These results confirm that Altair-LSFM provides the sub-cellular resolution, optical sectioning, and multi-color imaging performance necessary for quantitative biological imaging applications. To further assess performance in live specimens, we demonstrated dual-channel live-cell imaging. We modified the sample chamber to provide temperature control (Supplementary Note 2, Supplementary Figure 8) and imaged retinal pigment epithelial (RPE) cells with endogenously GFP and TagRFP-T tagged microtubules and vimentin, respectively (Supplementary Video 1, Figure 5). The cells exhibited robust motility, with time-lapse sequences revealing continuous, cell-side reorganization of microtubule and vimentin intermediate filaments throughout their bodies. Together, the fixed- and live-cell results establish that Altair-LSFM supports high-contrast, multi-color volumetric imaging at sub-cellular resolution in both static and dynamic cellular contexts.

## Discussion:

In this work, we demonstrated the viability of a baseplate-based approach for the dissemination a high-performance light-sheet microscope that is both accessible and straightforward to assemble by non-experts. By combining optical simulations, precision-machined component design, and experimental validation, we developed Altair-LSFM, a system that delivers sub-cellular resolution imaging with minimal alignment requirements. Characterization using fluorescent beads confirmed that Altair-LSFM achieves a resolution of 328, 330, and 464 nm before deconvolution, which improves to ~235 and 350 nm after deconvolution, in XY, and Z, respectively. These values are on par with the original, open-source version of LLSM (~230 and 370 nm, in XY, and Z, respectively^32^), confirming that our approach achieves state-of-the-art performance but in a streamlined, cost-effective, and optically less complex format. For example, the open-source LLSM illumination path includes approximately 29 optical components, each requiring precise lateral, angular, and coaxial alignment and maintenance. In contrast, Altair-LSFM contains only nine such elements. By this metric, Altair-LSFM is considerably simpler to assemble and maintain, further supporting our overarching goal of making high-resolution LSFM systems accessible to non-specialist laboratories. Nonetheless, it is worth noting that the original LLSM system offered a greater number of illumination modes (e.g., square, hexagonal, and structured illumination) and supported imaging in both a sample- and light-sheet scanning configurations (Supplementary Note 3). A complete parts list, and estimated cost, are provided in Supplementary Tables 1 and 2, respectively, offering a transparent roadmap for users looking to adopt and build the system. To aid planning, Supplementary Table 5 summarizes expected build and validation times, stratified by prior experience with optical system assembly and operation.

A number of open-source designs have also extended LSFM into the sub-cellular regime, including implementations of LLSM and diSPIM. These platforms have been instrumental in advancing dissemination and training within the imaging community, though they typically require routine optical alignment and operation by dedicated personnel. Commercial LLSM instruments from 3i and ZEISS have increased the availability of LLSM and have contributed substantially to disseminating this technology. According to vendor materials, the ZEISS variant of LLSM provides automated operation and long-term stability, providing a user-friendly, turn-key solution for sub-cellular imaging. The system’s design, which incorporates a meniscus lens to enable oblique imaging through a coverslip, simplifies setup and usability, albeit with a modest reduction in achievable resolution (reported deconvolved resolution: ~290 × 450–900 nm) relative to the original LLSM implementation and Altair-LSFM. However, acquisition and service costs remain high, and system modification by end users is limited. Altair-LSFM addresses a different need: an openly documented, modifiable, and reproducible path to sub-cellular LSFM that non-specialist laboratories can build, customize, and maintain at a fraction of the cost of a commercial system.

Building on this successful prototype, future efforts will focus on expanding both imaging capabilities and user experience. One natural evolution of this approach is the development of more advanced light-sheet microscope designs, such as ASLM^12,33^ and OPM^13,34^, which offer additional flexibility for imaging a broader range of sample types. Notably, OPM avoids the challenges associated with non-standard sample mounting and, owing to its single objective architecture, is fully compatible with standard environmental chambers used for live-cell imaging. Another avenue for improvement is the optimization of Altair-LSFM for cleared-tissue imaging, further extending its applicability into tissue contexts. Additionally, we aim to eliminate the need for an external analog output device, consolidating all triggering and waveform generation within a single unified controller, which will further reduce hardware dependencies and enhance system efficiency.

Another important consideration is the long-term scalability and routine maintenance of Altair-LSFM in a variety of lab environments. Multi-site benchmarking and community feedback will be pivotal to ensure consistent, reproducible performance across different setups. To aid planning, we also provide guidance on data storage in Supplementary Note 4. Future enhancements—such as self-alignment routines—could further boost imaging quality and throughput. To accelerate widespread adoption, we have thoroughly documented the entire assembly process in our GitHub repository^35^, which is also provided as a Supplementary Document. Although the construction of custom optical systems may seem intimidating to non-experts, our dissemination strategy draws inspiration from other successful open-source projects such as mesoSPIM, which has seen widespread adoption, including >30 implementations worldwide, through a similar model of exhaustive documentation, open-source control software, and community support via user meetings and workshops. For expert users who wish to tailor the instrument, we also provide all Zemax illumination-path simulations and CAD files, along with step-by-step optimization protocols, enabling modification and re-optimization of the optical system as needed. These customization resources are intended for users with prior experience in optical and optomechanical design, while the default configuration remains turnkey for non-experts.

A persistent challenge in advanced microscopy is the lengthy interval—from instrument conceptualization to commercialization—often spans close to a decade. By fostering open collaboration and continuous software-hardware integration, we envision Altair-LSFM evolving into a robust, ever-improving platform, well-suited to meet future research challenges in high-resolution, volumetric imaging. Integrating these systems with *navigate*^29^, we aim to further democratize intelligent imaging workflows and broaden the reach of cutting-edge instrumentation (Supplementary Note 5). With high-NA imaging, reduced mechanical and optical complexity, and lower cost, Altair-LSFM stands poised to accelerate LSFM adoption, delivering a powerful yet accessible solution for researchers immediately seeking access to high-resolution, cutting-edge, volumetric imaging.

## Materials and Methods

### Acquisition and Simulation Computer.

All microscope control and optical simulations were performed on a Colfax SX6300 workstation, configured to handle high-speed data acquisition and processing. It is equipped with dual Intel Xeon Silver 4215R processors (8 cores, 16 threads, 3.2 GHz), 256 GB of DDR4 3200 MHz ECC RAM, a 7.68 TB Samsung PM9A3 NVMe SSD for high-speed data acquisition, a 20 TB Seagate Exos X20 HDD for long-term data storage, a PNY NVIDIA T1000 4 GB GPU, and an Intel X710-T2L dual-port 10GbE.

### Cell Culture.

All cells were maintained at 37°C in a humidified incubator with 5% CO₂ and cultured in a 12-well plate on 5 mm cover slips, pre-rinsed with 70% ethanol. Mouse embryonic fibroblasts (MEFs) were cultured in Dulbecco’s Modified Eagle’s Medium (DMEM, Gibco) supplemented with 10% fetal bovine serum (FBS, Gibco) and 100 μg/mL penicillin-streptomycin. RPE-hTERT Vimentin-GFP/mTubulin-RFP cells were generated by TALEN-based genome editing^36^ and grown in ATCC-formulated DMEM/F12 supplemented with 10% FBS and 1% antibiotic–antimycotic. For imaging, cells were seeded on 5-mm round #0 coverslips placed in 6-well plates. During imaging, the coverslip was secured in a chamber and bathed in prewarmed media. Specifically, U2OS cells were imaged in FluoroBrite DMEM (Thermo Fisher) containing 5% FBS and 1% Anti-Anti, and RPE hTERT cells were imaged in DMEM/F12 medium without phenol red, supplemented with 5% FBS and 1% Anti-Anti. All imaging procedures were performed at 37 °C

### Immunofluorescence.

MEFs were cultured to approximately 50% confluency before processing. Cells were first rinsed with pre-heated (37°C) 1x Phosphate-Buffered Saline (PBS), and then briefly permeabilized and fixed with pre-heated PEM buffer [80 mM PIPES, 5 mM EGTA, 2 mM MgCl_2_, (pH: 6.8)], supplemented with 0.3% Triton-X and 0.125% glutaraldehyde (GA) for 30 seconds. A secondary fixation was then performed in preheated PEM buffer containing 2% paraformaldehyde (PFA) for 15 minutes at 37 °C. Following fixation, cells were washed three times with 1x PBS (2 minutes each). Unless otherwise indicated, all subsequent incubations were performed at room temperature with constant agitation. Residual aldehydes were quenched using 5 mM glycine for 10 minutes, after which the cells were blocked for 1 hour in 3% bovine serum albumin (BSA) and 0.01% Triton-X in 1x PBS. For indirect immunofluorescence, cells were incubated overnight at 4°C with primary antibodies diluted in staining buffer [1% BSA + 0.01% Triton-X in 1x PBS]: mouse anti-α-Tubulin (Sigma-Aldrich, #T9026, 1:250) and rabbit anti-GOLGA/GM130 (Proteintech, #11308–1-AP, 1:500). After three washes with PBST (1x PBS containing 0.01% Triton-X, 2 minutes each), cells were incubated for 1 hour with secondary antibodies diluted in staining buffer: donkey anti-mouse CF488A (Biotium, #20014–1, 1:500) and donkey anti-rabbit AlexaFluor 647 (Thermo Fisher Scientific, #A-31573, 1:500). Actin filaments were stained with phalloidin-CF568 (Biotium, #00044, 1:50) in 1x PBS for 1 hour. Finally, cells were incubated in DAPI nuclear dye (Thermo Fisher Scientific, #62248, 300 nM) in 1x PBS for 10 min. Samples were stored at 4°C in 1x PBS with 0.02% sodium azide (NaN_3_) until imaging.

### Preparation of Fluorescent Bead Samples.

A 5 mm glass coverslip was placed inside a petri dish, and approximately 100 μL of (3-Aminopropyl)triethoxysilane (APTS) at a concentration of 5 mM was applied to its surface. The APTS was incubated for 10–30 minutes to promote bead adhesion, after which the coverslip was lightly washed three times with deionized water. Fluorescent beads (Fluoresbrite YG Microspheres 0.10 μm, Polyscience, Inc) were diluted to the desired concentration (typically 10^−3^ or 10^−4^ for a normal distribution, 10^−6^ for a sparse distribution) and applied to the treated coverslip, where they were incubated for 2–20 min, with longer incubation times increasing bead density. Finally, the coverslip was lightly washed with deionized water to remove unbound beads before imaging.

### Image Deskewing.

After image acquisition, deskewing was performed to correct for the non-orthogonal scanning geometry between the piezo stage and the optical axes of the microscope. A custom Python script available on our GitHub repository, was used to perform deskewing. The user provides the path to the image stack along with the relevant imaging parameters: z-step size, xy pixel size, and the deskew angle. In our configuration, the deskew angle corresponds to 90°–60.5°, where 60.5° represents the angle between the normal of the sample mount and the microscope’s y-axis.

### Image Deconvolution.

Deconvolution was performed using PetaKit5D^37^ with standard operating parameters. All datasets were processed with a background level set to 100, and two iterations of the Optical Transfer Function Masked Wiener (OMW) deconvolution algorithm were applied. The Wiener parameter (alpha) was set to 0.005, the Optical Transfer Function Cumulative Threshold to 0.6, and the Hann window bounds to 0.8–1. No edge erosion was applied, and the resulting data were saved as 16-bit images. PSFs were simulated using the PSFGenerator^38^ package according to the illumination wavelength and numerical aperture of the light sheet, and the emission wavelength and numerical aperture of the detection objective. PSFs were modeled using the Richards & Wolf 3D Optical Model with a refractive index of 1.3333.

### Custom Machining and Fabrication.

All metal components were machined from 6061-T6x aluminum by Protolabs or Xometry, adhering to standard machining tolerances of ±0.005 inches. The original sample chamber was 3D printed using a Formlabs Form 3B resin printer with standard black resin. Our live-cell sample chamber was machined from 6061-T6x aluminum from Xometry. CAD files for all custom parts and procedures on how to place an order from Xometry using them are provided as a supplementary file, with up-to-date versions available on GitHub.

## Supplementary Material

Supplement 1

Supplement 2

## Figures and Tables

**Table 1.** LSFM variants and their associated illumination and detection optics. The table lists the type of microscope, the illumination and detection optics-including NA where available and immersion type in parentheses-as well as the overall design architecture (e.g., rail carrier, cage system, etc.).

Microscope	Illumination Optics	Detection Optics	Design
OpenSPIM^18^	Olympus UMPLFLN 10XW NA 0.3 (Water)	UMPLFLN 20XW NA 0.5 (Water)	Rail Carrier
X-OpenSPIM^17^	Nikon CFI Plan Fluor 10X NA 0.3 (Water)	Nikon CFI Apochromat NIR 40X W NA 0.8 (Water)	Rail Carrier
EduSPIM^16^	Zeiss LSFM 5X NA 0.1 (Air)	Zeiss LD Epiplan 5X NA 0.13 (Air)	Cage System
OpenSPIN^19^	Nikon CFI Plan Fluor 4X NA 0.13 (Air) Nikon Plan Fluor 10X NA 0.3 (Air)	Nikon CFI Plan Fluor 4X NA 0.13 (Air) Nikon CFI75 LWD 16X NA 0.8 (Water)	Rail Carrier
UC2^14^	Generic 4X NA 0.14 (Air)	Generic 4X NA 0.14 (Air) Generic 10X NA 0.3 (Air)	3D Printed Components
pLSM^15^	Mitutoyo Plan Apo 10X NA 0.28 (Air)	Mitutoyo Plan Apo 10X NA 0.28 (Air) ASI 54-10-12 16.67X NA 0.4 (Multi-Immersion)	Cage System
descSPIM^39^	500- and 150-mm cylindrical lenses (Air)	Thorlabs TL2X-SAP 2X NA 0.1 (Air)	Cage System
mesoSPIM^21^	Nikon 50 mm f/1.4 G (Air)	Olympus MVPLAPO 1X NA 0.15 (Air)	Rail Carrier
BT-mesoSPIM^20^	Nikon 50 mm f/1.4 G (Air)	Variable. Magnification 2–20X, NA 0.1–0.28 (Air)	Cage System
diSPIM^11^	Nikon CFI Apochromat NIR 40X W NA 0.8 (Water)	Nikon CFI Apochromat NIR 40X W NA 0.8 (Water)	Dovetail Tube System
CompassLSM^40^	Olympus XLFLUOR 4x NA 0.28 (Air/Water)	Olympus MVX PLAPO 1x NA 0.25 (Air) Olympus MVX PLAPO 2XC NA 0.5 (Air) Olympus UPlanFL 4x NA 0.13 (Air) Nikon CFI Plan Apo 10XC NA 0.5 (Water)	Rail Carrier
Lattice Light-Sheet Microscopy (LLSM)^8^	Special Optics 54-10-7 28.6X NA 0.67 (Water)	Nikon CFI75 Apochromat 25XC NA 1.1 (Water)	Free Space Optics
Altair-LSFM	Thorlabs TL20X-MPL 20X NA 0.6 (Water)	Nikon CFI75 Apochromat 25XC NA 1.1 (Water)	Custom Baseplate & Dovetail Tube System
