# Supplementary material for: Altair-LSFM A High-Resolution, Easy-to-Build Light-Sheet Microscope for Sub-Cellular Imaging
Source: bioRxiv. 2025 Nov 7:2025.04.08.647739. Preprint. [Version 3] doi: 10.1101/2025.04.08.647739 (PMC12637468; doi:10.1101/2025.04.08.647739)
Supplement: Supplement 2 [file NIHPP2025.04.08.647739v3-supplement-2.pdf]

bioRxiv preprint doi: https://doi.org/10.1101/2025.04.08.647739; this version posted November 7, 2025. The copyright holder for this preprint (which was not certified by peer review) is the author/funder, who has granted bioRxiv a license to display the preprint in perpetuity. It is made available under aCC-BY 4.0 International license.

SUPPLEMENTARY FIGURES

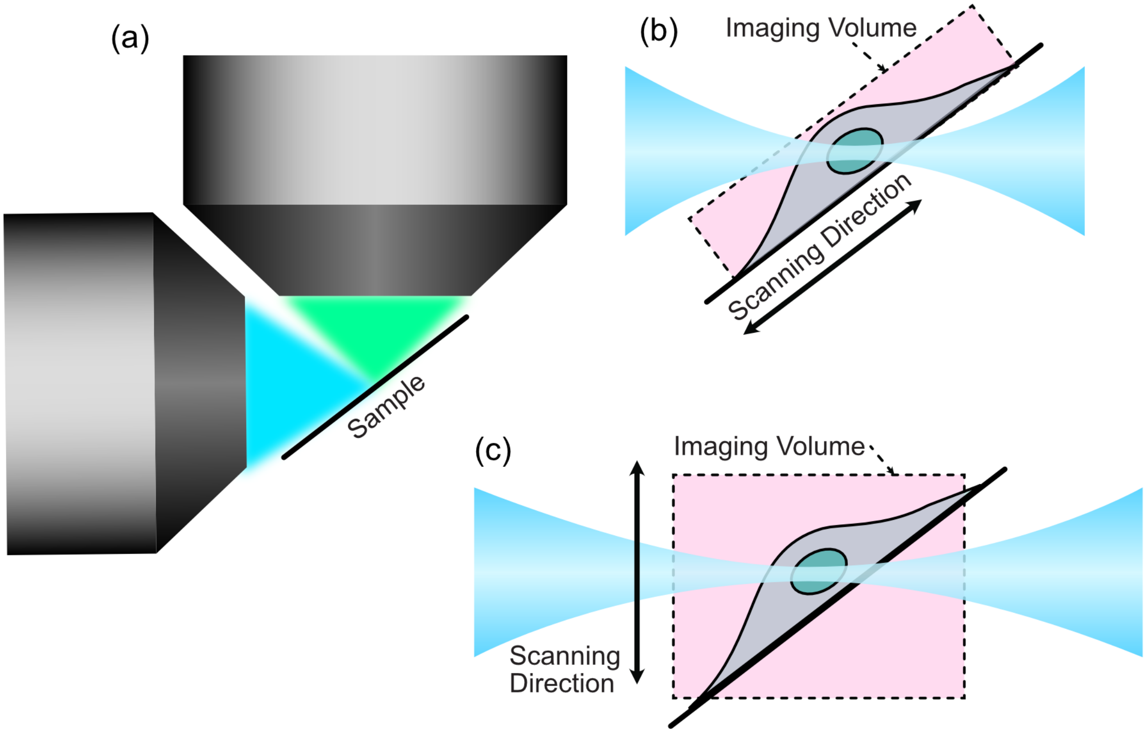

Supplementary Figure 1. Comparison of sample- and light-sheet-scanning modes. (a) In high-resolution light-sheet microscopy, the specimen must be positioned precisely at the intersection of the illumination and detection objective focal planes. To minimize aberrations in the excitation (blue) and detection (green) light paths, the specimen must be mounted at an angle that prevents marginal rays from interacting with the coverslip. In this configuration, only a narrow cross-section of an adherent cell is illuminated. (b) With a sample-scanning approach, the illumination beam requires a propagation length just sufficient to cover the thickest portion of the specimen—typically the nucleus—at the angle defined by the coverslip. For Altair-LSFM, the sample is mounted at ~30°, so a 6 µm-thick nucleus requires a beam propagation length of 6 µm / sin 30° ≈ 12 µm, which can be achieved with an illumination NA of ~0.285, producing a beam thickness of ~1 µm. The acquired volume is indicated by the dashed outline. (c) In contrast, a light-sheet-scanning configuration—where the light sheet and detection objective are synchronously translated in z—must generate a beam long enough to span the full cell diameter. For an adherent cell ~30 µm in diameter, the sheet must extend 30 µm / sin 30° ≈ 60 µm, requiring an illumination NA of ~0.128 and yielding a sheet thickness of ~2.3 µm. Together, these schematics illustrate how sample scanning enables the use of shorter, thinner light sheets that improve axial resolution while maintaining uniform illumination. The illumination NA required to achieve a given beam propagation length was estimated using the PSFGenerator package[38].

bioRxiv preprint doi: https://doi.org/10.1101/2025.04.08.647739; this version posted November 7, 2025. The copyright holder for this preprint (which was not certified by peer review) is the author/funder, who has granted bioRxiv a license to display the preprint in perpetuity. It is made available under aCC-BY 4.0 International license.

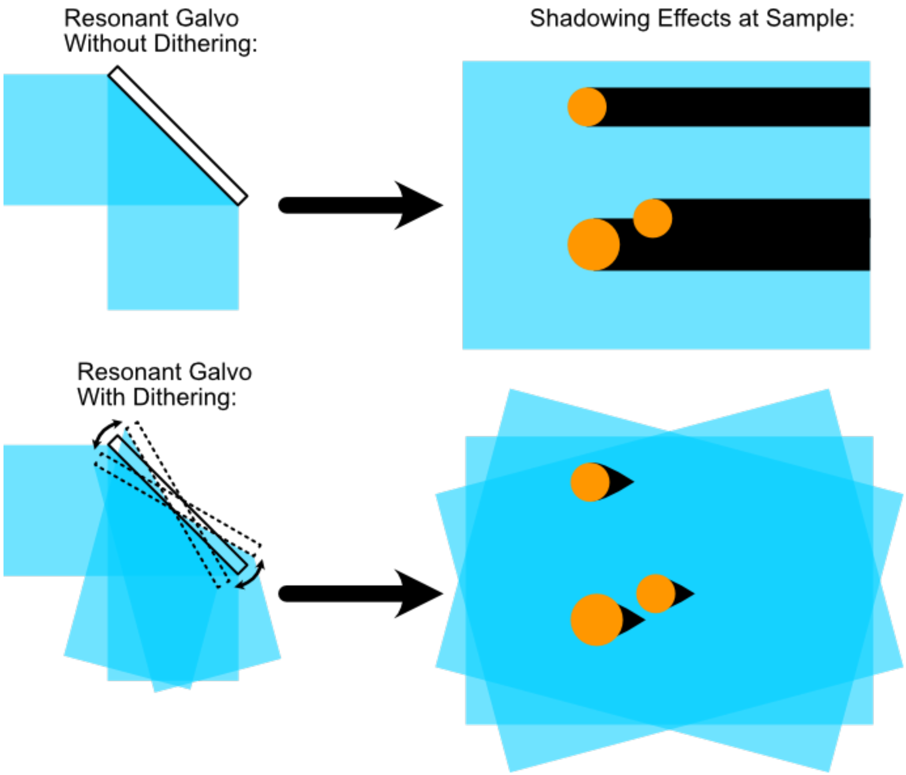

Supplementary Figure 2. Educational illustration depicting the conceptual function of the resonant galvo unit. Without pivoting of the light-sheet, objects within a sample can cast shadows due to scattering or refraction. When the resonant galvo is engaged, the illumination sheet rapidly pivots at a frequency of 4 kHz such that the sample is effectively illuminated from multiple directions, and the shadows are correspondingly displaced. As a result, light effectively “reaches around” objects, and the shadows are averaged out over the image acquisition period. This figure is intended as a conceptual aid to help readers visualize the role of the resonant galvo rather than act as a quantitative representation.

bioRxiv preprint doi: https://doi.org/10.1101/2025.04.08.647739; this version posted November 7, 2025. The copyright holder for this preprint (which was not certified by peer review) is the author/funder, who has granted bioRxiv a license to display the preprint in perpetuity. It is made available under aCC-BY 4.0 International license.

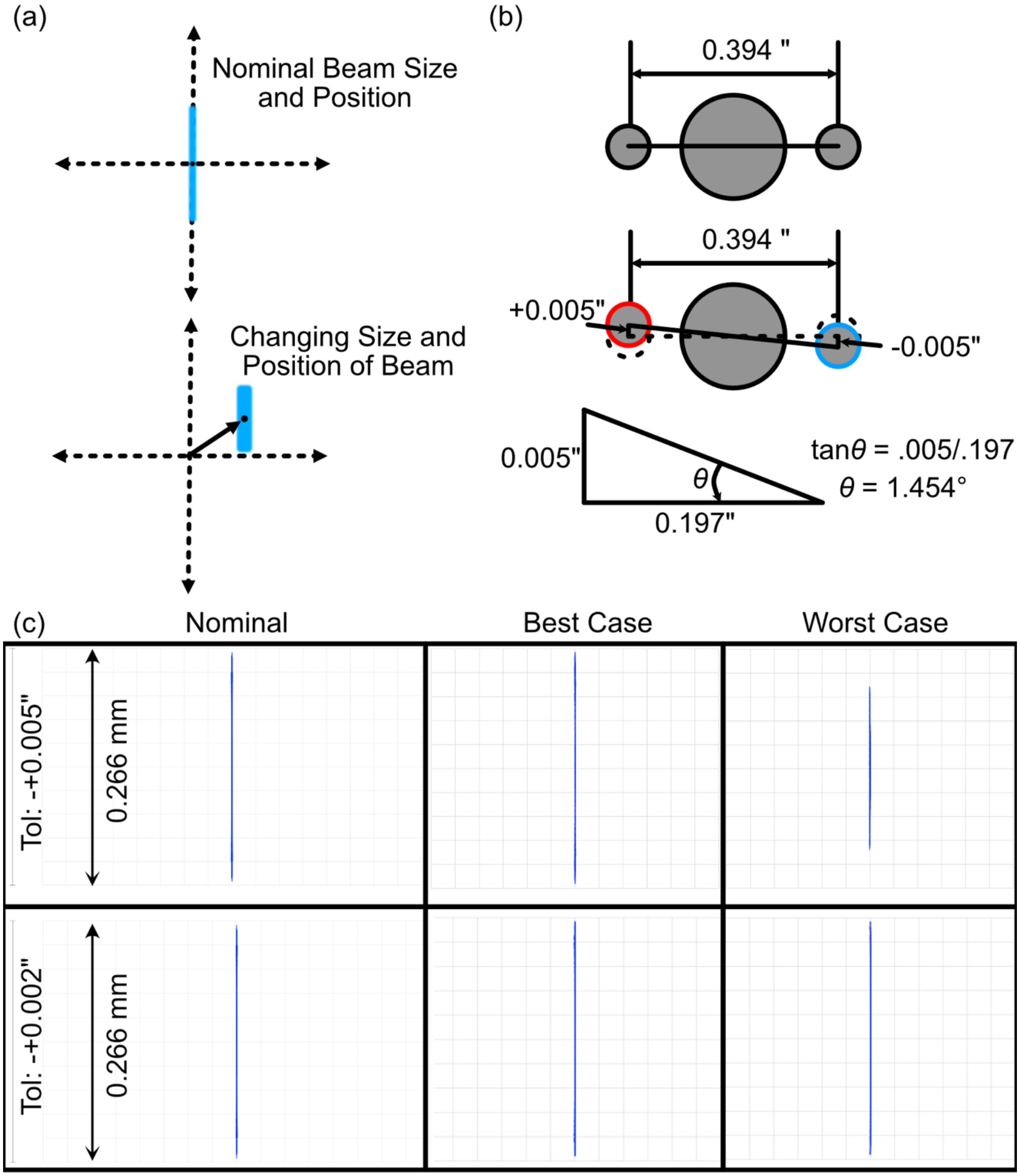

Supplementary Figure 3. (a) Depiction of the merit function criteria used in our tolerance analysis, where we observed how the beam profile in the perturbed instances changes in both size and position. (b) Schematic of the Polaris dowel pin mounting configuration when considering machining tolerances, where in a worst-case scenario the angle offset would be 1.454 degrees. (c) Nominal, best case, and worst-case beam profiles in xz for both coarse (+-0.005", top row) and fine (+-0.002", bottom row) machining tolerances.

bioRxiv preprint doi: https://doi.org/10.1101/2025.04.08.647739; this version posted November 7, 2025. The copyright holder for this preprint (which was not certified by peer review) is the author/funder, who has granted bioRxiv a license to display the preprint in perpetuity. It is made available under aCC-BY 4.0 International license.

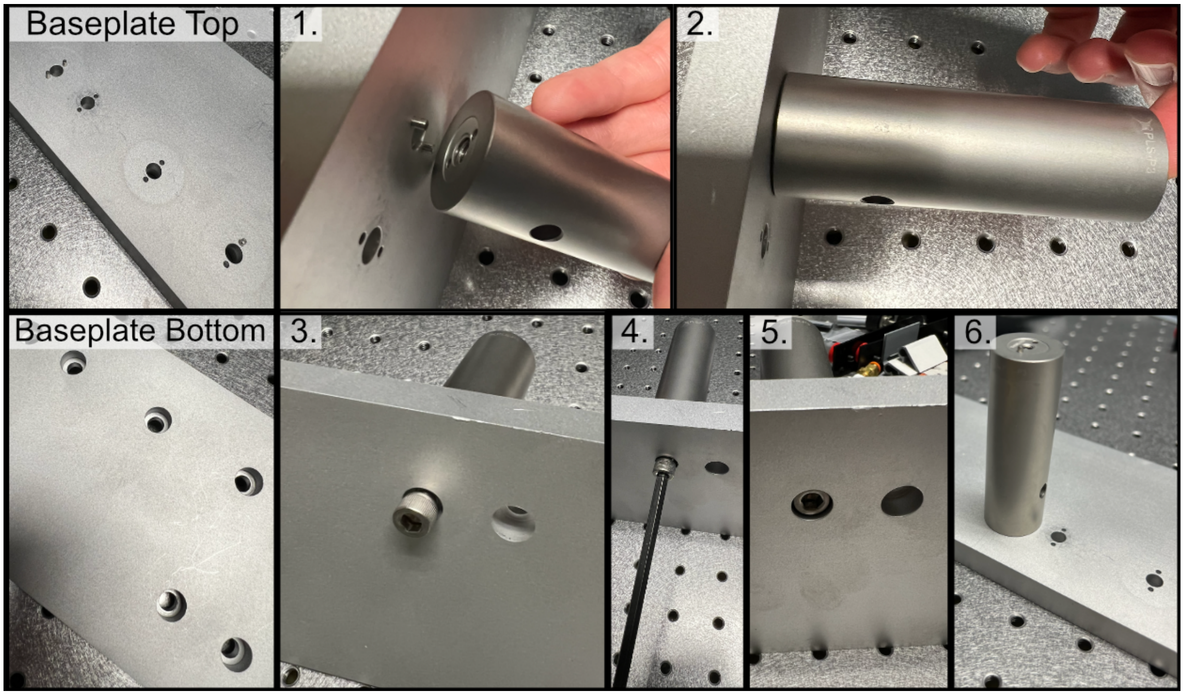

Supplementary Figure 4. Process of affixing a post to the baseplate, where one first places the post onto dowel pins inserted into the corresponding holes and then fixes the post to the baseplate with a screw.

bioRxiv preprint doi: https://doi.org/10.1101/2025.04.08.647739; this version posted November 7, 2025. The copyright holder for this preprint (which was not certified by peer review) is the author/funder, who has granted bioRxiv a license to display the preprint in perpetuity. It is made available under aCC-BY 4.0 International license.

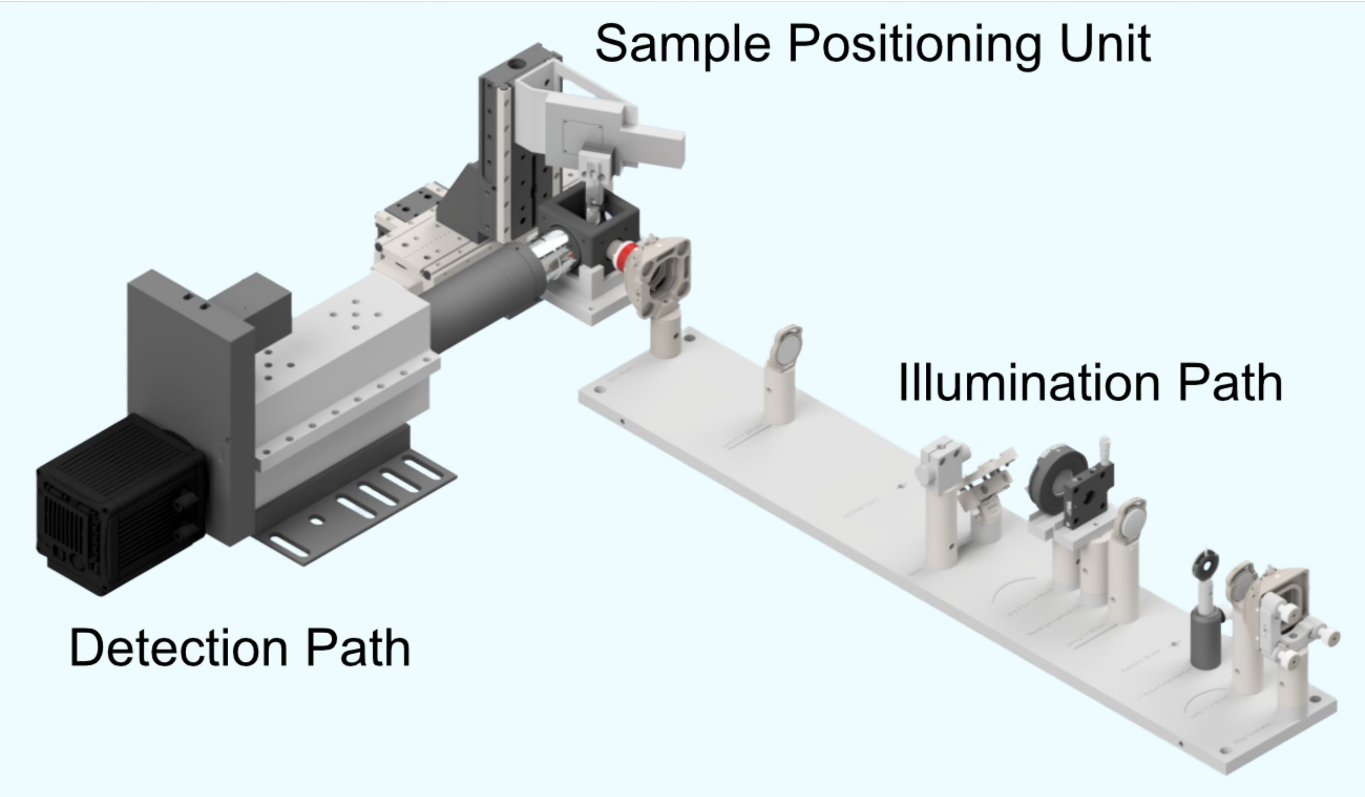

Supplementary Figure 5. CAD rendering of our full system consisting of an illumination path, a detection path, and a dedicated sample positioning assembly.

bioRxiv preprint doi: https://doi.org/10.1101/2025.04.08.647739; this version posted November 7, 2025. The copyright holder for this preprint (which was not certified by peer review) is the author/funder, who has granted bioRxiv a license to display the preprint in perpetuity. It is made available under aCC-BY 4.0 International license.

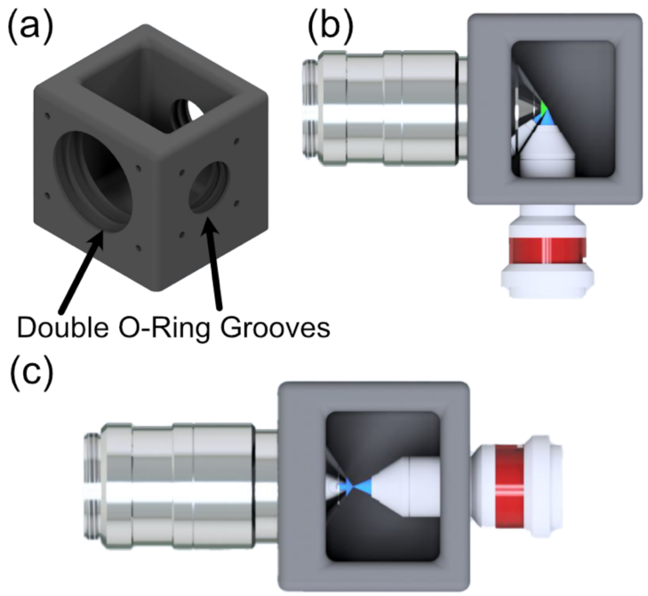

Supplementary Figure 6. (a) CAD rendering of our custom sample chamber, featuring three possible objective ports, each with two sets of O-rings to ensure a liquid-proof seal. (b) Top-down rendering of the traditional imaging configuration for the system, where the illumination and detection objectives are placed orthogonal to one another. (c) The second transmissive imaging configuration of the system used to image the beam itself, where the illumination objective is place directly in front of the detection objective.

bioRxiv preprint doi: https://doi.org/10.1101/2025.04.08.647739; this version posted November 7, 2025. The copyright holder for this preprint (which was not certified by peer review) is the author/funder, who has granted bioRxiv a license to display the preprint in perpetuity. It is made available under aCC-BY 4.0 International license.

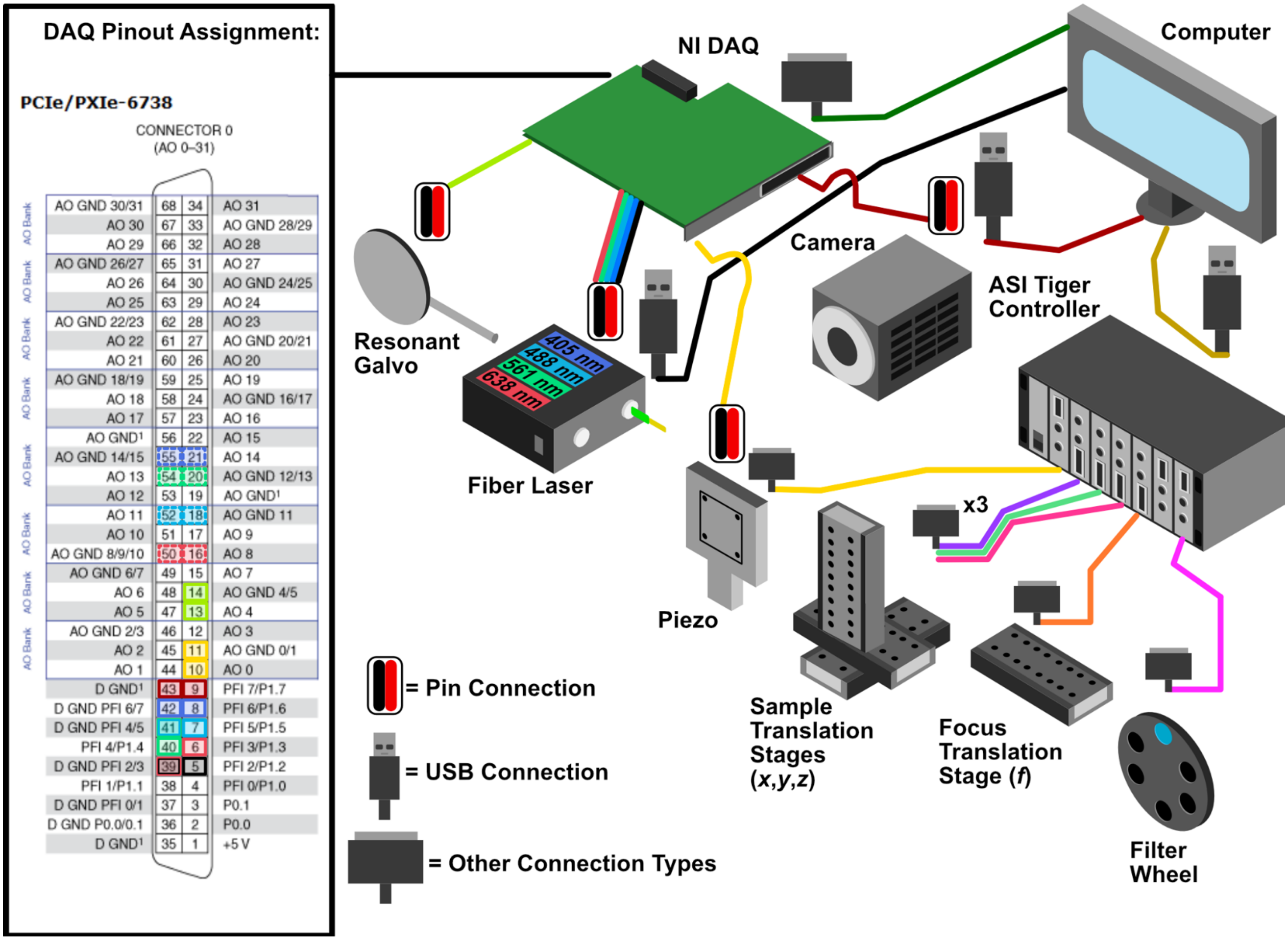

Supplementary Figure 7. General wiring diagram of the system showing all the optoelectrical and optomechanical components used, and an inset showing how these components are wired into the NI DAQ.

bioRxiv preprint doi: https://doi.org/10.1101/2025.04.08.647739; this version posted November 7, 2025. The copyright holder for this preprint (which was not certified by peer review) is the author/funder, who has granted bioRxiv a license to display the preprint in perpetuity. It is made available under aCC-BY 4.0 International license.

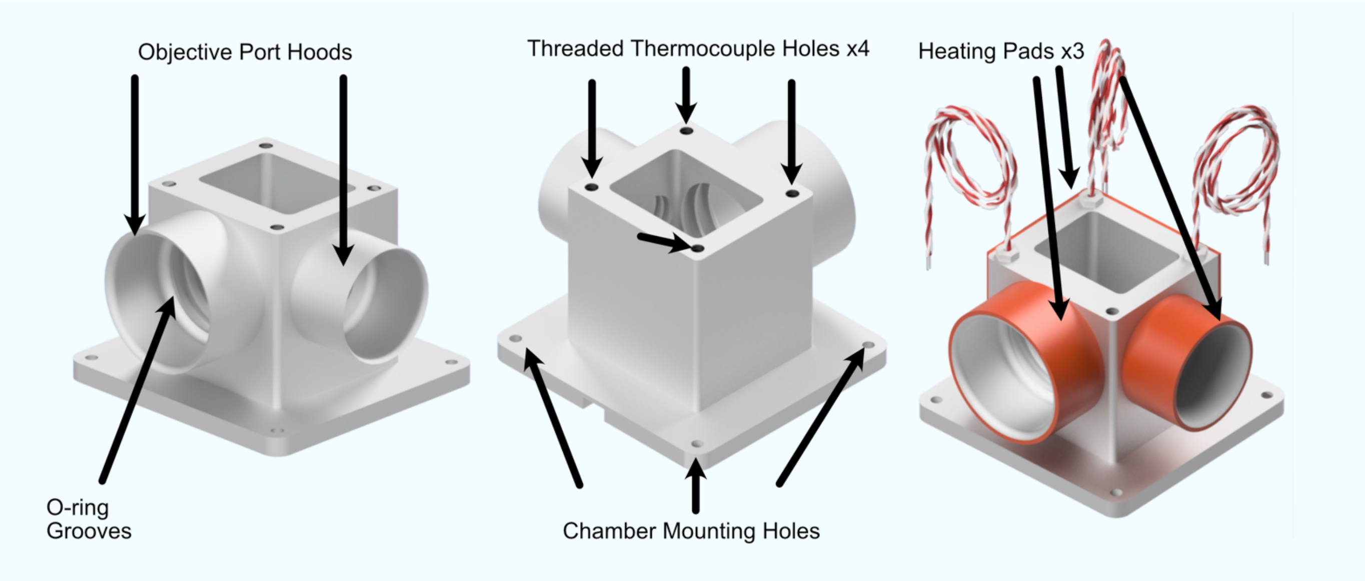

Supplementary Figure 8. CAD renderings of our custom heated sample chamber design. This updated design utilizes thermocouples and heating pad elements for temperature regulation, enabling live-cell imaging capabilities for Altair-LSFM.

bioRxiv preprint doi: https://doi.org/10.1101/2025.04.08.647739; this version posted November 7, 2025. The copyright holder for this preprint (which was not certified by peer review) is the author/funder, who has granted bioRxiv a license to display the preprint in perpetuity. It is made available under aCC-BY 4.0 International license.

SUPPLEMENTARY VIDEOS

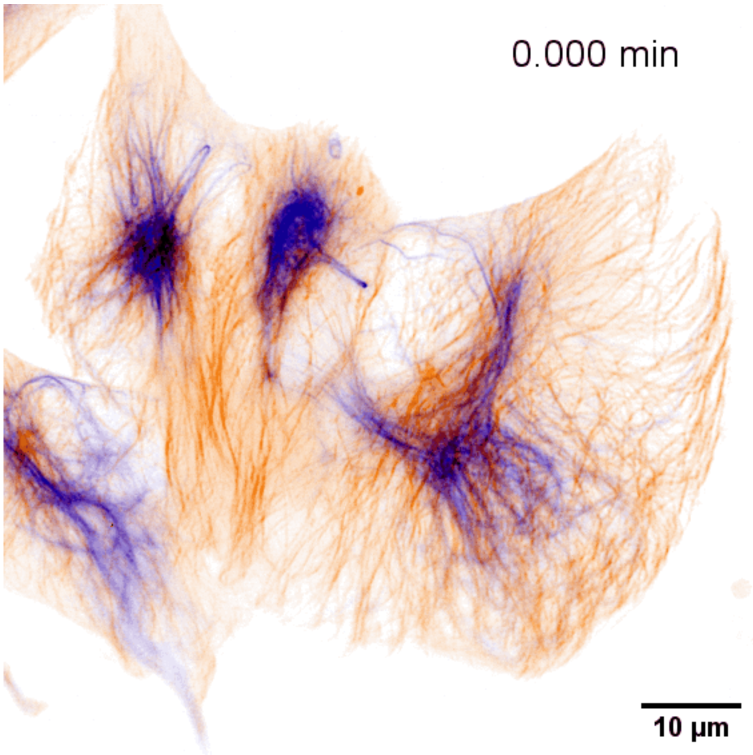

Supplementary Video 1. Representative frame from a time-lapse of four RPE-hTERT cells with endogenously tagged cytoskeletal markers: microtubules (red) and vimentin (blue).

bioRxiv preprint doi: https://doi.org/10.1101/2025.04.08.647739; this version posted November 7, 2025. The copyright holder for this preprint (which was not certified by peer review) is the author/funder, who has granted bioRxiv a license to display the preprint in perpetuity. It is made available under aCC-BY 4.0 International license.

SUPPLEMENTARY TABLES

Name	Manufacturer	Description	Qty	Approximate Cost
**Shared Equipment**				
TG16-BASIC	ASI	Tiger Controller – 16 Bay System	1	$6,350
PCIe-6738	NI	Data Acquisition Card	1	$2,200
SCB-68A	NI	Noise Rejecting Terminal Block	1	$500
SHC68-68-A2	NI	Test Cable Assembly	1	$250
784-736-02R	TMC	36x60x18” Performance Series Optical Top	1	$6,275
14UD-42X-24	TMC	Ultradamp Vibration Isolation with Casters	1	$9,300
SX6300	Colfax International	Colfax SX6300 Workstation	1	$6,800
**Detection Path**				
LS-100-AMCCH	ASI	100 mm Linear Focusing Stage, 16 TPI	1	$2,250
TGDCM2	ASI	2-Axis Stage Control Card	1	$1,050
C60-EXT-15	ASI	15 mm Tube Extension	1	$75
RAO-0051	ASI	M32x0.75 Threaded Sleeve	1	$150
FW-0002-8	ASI	8-Position Filter Wheel	1	$3,500
FW-C-MNT-K1	ASI	Filter Wheel to MIM Adapter Kit	1	$400
C60-TUBE-400	ASI	400 mm Achromatic Tube Lens	1	$875
TGFW	ASI	Filter Wheel Control Card	1	$1,150
C13440-20CU	Hamamatsu	ORCA Flash4.0 V3	1	$16,500
OH-CAMRA-1007	Hamamatsu	Firebird CamLink Board	1	$2,000
N25X-APO-MP	Thorlabs	Nikon 25x NA 1.1 Detection Objective	1	$33,810
445/20-25nm	Semrock	Brightline Bandpass filter	1	$375
529/24nm	Semrock	Brightline Bandpass filter	2	$375
605/15-25nm	Semrock	Brightline Bandpass filter	1	$375
676/29-25nm	Semrock	Brightline Bandpass filter	1	$375
**Sample Positioning**				
LS-5012	ASI	Breadboard Adapter	1	$160
LS-5013	ASI	Right Angle Bracket	1	$250
DV-6010-C	ASI	Dovetail Mount Pair	1	$230
LS-50-AMCLLS	ASI	50 mm Linear Stage with Stainless Steel Slide	1	$2,425
LS-100-AMERL	ASI	100 mm Linear Stage, 16 TPI, Extended, Right	1	$3,100
LS-50-AMELL	ASI	50 mm Linear Stage	1	$2,250
TGADEPT	ASI	Piezo Control Card	1	$1,500
HS1.100	PiezoConcept	Piezo Concept 100 Micron Piezo Stage	1	$4,100
ADAPTHS1BB	PiezoConcept	Adapter Plate for PiezoConcept HS1	1	$120
Angle Bracket Adapter	Xometry	Angle adapter for mounting piezo at an angle	1	$592.58
**Illumination Path**				
L4CC	Oxxius	Multicolor Laser Source	1	25,000
P3-405B-FC-1	Thorlabs	Fiber Cable Single Mode FC/APC	1	$120

bioRxiv preprint doi: https://doi.org/10.1101/2025.04.08.647739; this version posted November 7, 2025. The copyright holder for this preprint (which was not certified by peer review) is the author/funder, who has granted bioRxiv a license to display the preprint in perpetuity. It is made available under aCC-BY 4.0 International license.

CFC11A-A	Thorlabs	Fiber Collimator	1	$370
Polaris-K1XY	Thorlabs	Kinematic Mount for Fiber Laser Collimator	1	$1375
AC254-030-A-ML	Thorlabs	Achromatic Lens f=30mm (L1)	1	$125
AC254-080-A-ML	Thorlabs	Achromatic Lens f=80mm (L2)	1	$115
ACY254-075-A	Thorlabs	Achromatic Cylindrical Lens f=75mm (L3)	1	$445
AC254-250-A-ML	Thorlabs	Achromatic Lens f=250mm (L4)	1	$90
PF10-03-P01	Thorlabs	1” Protected Silver Mirror	1	$55
TL20X-MPL	Thorlabs	Illumination Objective	1	$4820
VA100CP	Thorlabs	Rectangular Aperture	1	$315
IDA12	Thorlabs	Circular Aperture Iris	1	$60
Polaris-P150	Thorlabs	1” Diameter Polaris Mounting Post, 1.5” Length	1	$45
Polaris-P2	Thorlabs	1” Diameter Polaris Mounting Post, 2” Length	1	$45
Polaris-P3	Thorlabs	1” Diameter Polaris Mounting Posts, 3” Length	3	$45
Polaris-P075*	Thorlabs	1” Diameter Polaris Mounting Post, 0.75” Length	1	$45
Polaris-P225*	Thorlabs	1” Diameter Polaris Mounting Post, 2.25” Length	1	$45
Polaris-P250*	Thorlabs	1” Diameter Polaris Mounting Post, 2.5” Length	2	$45
Polaris-MA45	Thorlabs	Polaris 45 Degree Adapter for Mirror Mount	1	$50
Polaris-B1S	Thorlabs	Polaris Flexure-Closed Lens Mount	3	$110
Polaris-1XY	Thorlabs	Polaris XY Translation Mount for Illumination Objective	1	$1020
Polaris-K1S4	Thorlabs	Polaris 1” Mirror Mount	1	$185
SM1A12	Thorlabs	Thread Adapter for Illumination Objective into Polaris-1XY	1	$25
6SC04KA040-01Y	Novanta	1-Axis 4 kHz Resonant Galvo and Servo	1	$3,700
Galvo Holder	Protolabs	Holder for Galvo	1	$400
TGPOW-12-3	ASI	Galvo Low Noise Power Supply +12V 3A	1	$950
RSP1 Adapter	Xometry	Custom Adapter for Thorlabs RSP1 to Polaris Posts	1	$80
VA100CP Adapter	Xometry	Custom Adapter for Thorlabs VA100CP to Polaris Posts	1	$80
CP02 Adapter	Xometry	Custom Adapter for Thorlabs CP02 to Polaris Posts	1	$80
Illumination Path Baseplate	Xometry	Baseplate for Illumination Path	1	$1000
**Live-Cell Imaging**				
Custom Sample Chamber	Xometry	Custom Designed Live-Cell Sample Chamber	1	$530
1797N11	McMaster Carr	Benchtop Autotuning Temperature Controller, Type J Thermocouple, 1 Bay	1	$835
1797N103	McMaster Carr	Benchtop Autotuning Temperature Controller, Type J Thermocouple, 2 Bay	1	$1390
5586N11	McMaster Carr	3" Dual Thermocouple 1/8" Diameter Probe, Type J	1	$120
5843N12	McMaster Carr	1" Threaded Thermocouple 1/4"-20 Threading	1	$100
35765K373	McMaster Carr	2x5" Adhesive Backed Heat Sheet	1	$80
35475K362	McMaster Carr	5x1" Ultrathin Heat Sheet	1	$120
35765K367	McMaster Carr	6x1" Adhesive Backed Heat Sheet	1	$75

Supplementary Table 1. Detailed equipment list. Prices are approximate and subject to change. *Indicates that the part was custom ordered from Thorlabs.

bioRxiv preprint doi: https://doi.org/10.1101/2025.04.08.647739; this version posted November 7, 2025. The copyright holder for this preprint (which was not certified by peer review) is the author/funder, who has granted bioRxiv a license to display the preprint in perpetuity. It is made available under aCC-BY 4.0 International license.

Group	Approximate Cost
Illumination Path	$41,195
Sample Positioning	$14,727
Detection Path	$63,635
Shared Equipment	$31,675
Live-Cell Imaging	$3250
Total:	$154,482

Supplementary Table 2. Approximate cost.

Component	Analog/Digital	Ground Pin	Active Pin
Resonant Galvo	Analog	14	13
Piezo Motor	Analog	11	10
Camera	Digital	43	9
Fiber Laser (FL) Output Shutter	Digital	39	5
FL λ = 405 nm AO Port	Analog	55	21
FL λ = 405 nm IO Port	Digital	42	8
FL λ = 488 nm AO Port	Analog	18	52
FL λ = 488 nm IO Port	Digital	41	7
FL λ = 561 nm AO Port	Analog	20	54
FL λ = 561 nm IO Port	Digital	41	40
FL λ = 638 nm AO Port	Analog	50	16
FL λ = 638 nm IO Port	Digital	39	6

Supplementary Table 3. Electrical pinouts used on National Instruments PCIe-6738 data acquisition card. All analog and digital connections were made using a National Instruments SCB-68A shielded terminal block.

Exposure Time (ms)	Average Frame Rate (Hz)
10	62.5
50	17.6
100	9.2
200	4.8

Supplementary Table 4. Acquisition performance for a 50 µm z-stack acquired at 0.25 µm step size (200 frames; 2048 × 512 pixels per frame). The mean inter-frame dead time was 7.25 ms, of which ~1 ms arose from piezo stepping; the remainder was dominated by camera readout.

bioRxiv preprint doi: https://doi.org/10.1101/2025.04.08.647739; this version posted November 7, 2025. The copyright holder for this preprint (which was not certified by peer review) is the author/funder, who has granted bioRxiv a license to display the preprint in perpetuity. It is made available under aCC-BY 4.0 International license.

Experience	Illumination Path Assembly	Full System Assembly	Fine Alignment	Imaging & Validation
**Novice**	~1-3 hours	1-4 Days	1 Week	2 Weeks
**Moderate**	~1-3 hours	1-4 Days	1 Week	1 Week
**Expert**	~1 hour	1 Day	1-2 Days	1 Week

Supplementary Table 5. Approximate time considerations based on user experience level. A novice is defined as someone entirely new to optical systems, with no prior experience in their operation or alignment. A moderate user has some prior experience operating or using optical systems but limited experience assembling or aligning them. An expert user has substantial experience designing, building, and aligning custom optical systems and is therefore expected to complete setup and validation tasks more efficiently.

bioRxiv preprint doi: https://doi.org/10.1101/2025.04.08.647739; this version posted November 7, 2025. The copyright holder for this preprint (which was not certified by peer review) is the author/funder, who has granted bioRxiv a license to display the preprint in perpetuity. It is made available under aCC-BY 4.0 International license.

SUPPLEMENTARY NOTES

Supplementary Note 1. Design Rationale and Cost Considerations for Altair.

A core design objective of Altair-LSFM was to develop a high-performance, open-source light-sheet microscope that is accessible to a broad community of users, including those without extensive optical design or engineering experience. While we prioritized cost-effectiveness wherever possible, several design choices were made to balance performance, reliability, and ease of adoption, rather than minimizing cost alone. Moreover, we sought to minimize the complexity of sourcing, configuring, and integrating components from disparate vendors by favoring consolidated, multipurpose hardware. Despite this, end users are free to customize the hardware configuration to suit their experimental needs. Different detection objectives, stages, filter wheels, or cameras can be readily incorporated, as *navigate* natively supports a broad range of hardware devices. New hardware can also be easily integrated through *navigate's* modular device interface, enabling users to expand functionality or adapt the system to specific equipment without extensive reconfiguration.

The estimated total cost of a complete Altair-LSFM system in its default configuration, including the optical table and laser source, is approximately $150,000. Although this may still represent a barrier for some research groups, it is substantially lower than most commercial systems offering comparable sub-cellular resolution performance (e.g., LLSM systems from 3i or Zeiss, which are ~$600,000 and >$800,000 USD, respectively, or a diSPIM from ASI, which is ~$250,000). These values very substantially depending on system configuration and are provided only as approximate guides based on publicly available information, including instrumentation, published news articles, and personal communications. One major cost driver in LLSM-based systems is the use of a spatial light modulator, which is a relatively low-efficiency device and necessitates upgrading to higher-power laser sources[37] (e.g., ~500 mW). For Altair-LSFM, a full list of system components, vendors, and approximate costs is provided in **Supplementary Tables S1 and S2**. Below, we outline the primary cost drivers in the system, our rationale for selecting them, and potential alternatives that could be used in future variants to reduce cost, albeit by potentially compromising system performance.

<u>Illumination and Detection Objectives</u>

The illumination and detection objectives used in LSFM must be treated as a single, coupled optical system. For Altair-LSFM, the primary cost driver is the detection/illumination pair built around the Nikon N25X-APO-MP 25×/1.1 NA detection objective (≈ $33,000), which must be paired with either the Thorlabs TL20X-MPL 20×/0.6 NA illumination objective (≈ $5,000) or the Special Optics 54-10-7@488–910 nm (≈ $15,000). The Special Optics objective provides a slightly higher numerical aperture (0.66 NA) but a shorter working distance (3.74 mm) compared to the Thorlabs TL20X-MPL (0.6 NA, 5.5 mm). Both are optimized for water immersion and achromatically corrected across the visible spectrum. Using the vectorial Richards & Wolf 3D optical model, the expected illumination PSF at 488 nm and maximum NA is approximately 451 × 2240 nm (XY × Z) for the Special Optics 54-10-7 and 496 × 2711 nm for the Thorlabs TL20X-MPL. While the higher NA of the Special Optics lens marginally improves resolution, the increased working distance of the Thorlabs objective makes it compatible with alternative detection objectives and facilitates imaging with larger coverslips (see below). Moreover, in practical applications, the full numerical aperture of these objectives is rarely used, as doing so would require exceptionally thin specimens.

Users seeking to reduce cost may substitute the Nikon 25× with a Zeiss W Plan-Apochromat 20×/1.0 (≈ $7,000). This objective is compatible with the existing detection path (no different tube lens required; aberration corrections are internal to the primary objective) and, when paired with the TL20X-MPL for illumination, is expected to deliver similar practical performance while easing sample handling by allowing coverslips larger than 5 mm in diameter. A more aggressive cost reduction is to redesign the optical train around Nikon N40X-NIR 40×/0.8 objectives for both illumination and detection. However, because vendor ray files/Zemax models are not available for these lenses, such a redesign would require introducing additional alignment degrees of freedom and tolerancing steps, increasing assembly complexity. Moreover, the lower NA would reduce raw resolution to ~400 nm laterally and axially[12] and halve photon collection, which scales with NA^2, lowering sensitivity—tradeoffs that may be unacceptable for low-signal or fast volumetric imaging. While the combination of objectives used in this implementation of Altair-LSFM has a total list price of approximately $38,000, substituting the detection objective with the Zeiss W Plan-Apochromat 20×/1.0 would reduce this cost to ≈ $12,000, and a full redesign around Nikon 40×/0.8 objectives for both illumination and detection would further reduce the total to ≈ $6,000, providing a clear path toward more cost-effective configurations depending on experimental needs and available resources

bioRxiv preprint doi: https://doi.org/10.1101/2025.04.08.647739; this version posted November 7, 2025. The copyright holder for this preprint (which was not certified by peer review) is the author/funder, who has granted bioRxiv a license to display the preprint in perpetuity. It is made available under aCC-BY 4.0 International license.

Scientific Camera

Scientific CMOS (sCMOS) cameras are a major investment in Altair-LSFM. Older Hamamatsu ORCA-Flash 4.0 units could sometimes be obtained near ~$15k, whereas current-generation sCMOS from major vendors (Hamamatsu, Photometrics, PCO, etc.) typically fall in the $30k–$40k range. The cost is justified by characteristics that are especially important for light-sheet imaging: high quantum efficiency, low read noise with effective suppression of dark current, large sensors that enable highly parallel acquisition across a wide field, and fast readout rates that support high frame rates. In our configuration (50x effective magnification, 6.5 µm pixels), the camera provides Nyquist-sampled pixels (~130 nm) across a wide 25 mm sensor, which is critical for capturing ~150–200 planes per adherent cell per channel at biologically relevant speeds—particularly for live-cell experiments.

Lower-cost industrial CMOS cameras (e.g., Ximea MU196CR-ON, recently demonstrated in a Direct-View OPM[41] configuration) can in principle substitute for sCMOS in budget-constrained builds. However, in our experience these sensors exhibit higher noise floors and reduced dynamic range, which limits sensitivity in low-signal regimes. They are also typically slower than leading sCMOS cameras and show greater variability in fixed-pattern defects (e.g., hot pixels), often necessitating calibration and post-processing. For applications that demand sub-cellular resolution at high speed and low illumination, we therefore recommend sCMOS as the default choice; industrial CMOS can be considered for cost-reduced variants with the understanding that overall performance will be compromised (sensitivity, speed, and image uniformity).

ASI Equipment

Altair-LSFM consolidates operation of all motion axes and the emission filter wheel into a single ASI Tiger Controller, including motorized XYZ sample positioning, a high-speed piezo Z scan for volumetric acquisition, and a motorized focus (F) axis for precise co-focusing of the detection plane. The complete system—XYZ and F stages, piezo stage, motorized emission filter wheel, ASI Tiger controller, and basic optomechanical adapters—totals ~$31,000 (~20% of system cost).

Cost-reduced variants are possible, but each entails performance or complexity trade-offs. Replacing motorized XYZ/F with manual stages can save ~$12,000, but removes critical capabilities such as autofocus, 3D tiling, and multi-position acquisition. Eliminating the piezo and relying solely on linearly encoded Z stages lowers upfront cost and complexity, yet significantly increases repositioning time and reduces volumetric throughput, which is especially limiting for live-cell imaging. Worth noting, our sample scanning piezo could deliver higher bandwidth with the vendor's dedicated controller; however, we favor a unified Tiger Controller-based control stack to minimize the number of controllers to wire, configure, and maintain, thereby streamlining adoption for non-experts.

Open-source mechanical platforms (e.g., OpenFlexure[42]) could, in principle, be adapted to further reduce cost, but would require custom assembly and software integration—shifting burden to the end user—and may exhibit reduced accuracy/precision and increased hysteresis compared with the closed-loop stages used here. Similarly, the motorized emission filter wheel can be omitted in favor of a fixed multi-band emission filter (saving ~$5,000), but this increases spectral crosstalk and often necessitates post-acquisition spectral unmixing, complicating workflows and potentially reducing sensitivity for dim samples.

Analog and Digital Control

Altair-LSFM uses a National Instruments (NI) 6738 (PXIe/PCIe form factor) for real-time analog and digital control. The board provides 32 analog outputs with 16-bit DAC resolution at up to 1 MS/s update rate, which we use to generate waveforms for the resonant/galvo drive, piezo position control, and analog laser intensity. The card also exposes 10 digital I/O lines that we use for laser modulation (TTL) and camera triggering/synchronization. We selected this platform because it is readily available internationally, offers robust, deterministic timing, and comes with long-term driver support and tooling that reduces integration burden across operating systems and labs. In future releases, we aim to eliminate the NI card by consolidating triggering and waveform generation into the ASI Tiger Controller, further reducing hardware count, wiring, and software dependencies while keeping the system turnkey for non-experts. Importantly, we opted to avoid lower-cost microcontroller (MCU) solutions (e.g., USB microcontrollers with external DACs) because they carry several practical disadvantages:

- Timing determinism and jitter: General-purpose MCUs and USB links typically exhibit µs–ms-scale jitter and non-deterministic latency, complicating tight synchronization between camera exposure, galvo phase, laser blanks, and piezo motion.
- Waveform fidelity and throughput: Many MCUs cannot sustain simultaneous, multi-channel DAC streaming at 1 MS/s.

bioRxiv preprint doi: https://doi.org/10.1101/2025.04.08.647739; this version posted November 7, 2025. The copyright holder for this preprint (which was not certified by peer review) is the author/funder, who has granted bioRxiv a license to display the preprint in perpetuity. It is made available under aCC-BY 4.0 International license.

- Noise and analog performance: MCU-centric solutions often have higher analog noise, poorer calibration/drift, and require custom electrical interfaces.
- Scalability and maintenance: NI provides stable drivers, diagnostics, and a clearer path for multi-OS support.
- Feature trade-offs: Replacing the NI card would push more complexity into software/firmware and wiring.

Supplementary Note 2. Environment Chamber Design.

To support live-cell imaging with Altair-LSFM, we developed a temperature-controlled sample chamber (**Figure S8**). Typical cell viability temperatures span ~25 °C for yeast to 37 °C for mammalian cells. The chamber integrates adhesive heating pads and embedded thermocouples in the chamber wall, regulated by a TempCo controller to maintain a fixed setpoint. Relative to our initial design, we removed the secondary illumination port (formerly used for linear light-sheet imaging), which allowed us to mount larger heaters along two exterior walls for more uniform heating of the chamber volume. To minimize thermal gradients— which can induce drift or optical aberrations (e.g., with the Nikon 25× objective), we also provide indirect objective heating. Two add-on hoods surround the illumination and detection ports; each wrapped with heating pads. In total, the system heats three zones: (1) illumination-objective hood, (2) detection-objective hood, and (3) the two external walls opposite the objective ports. All off-the-shelf components (outside the custom chamber) were sourced from McMaster-Carr; the total live-cell upgrade cost was ~$3,250 (**Table S2**).

Commercial environmental chambers often maintain 5% CO_2 (stabilizing pH in bicarbonate-buffered media) and add humidification to limit evaporation; turnkey systems of this type typically cost on the order of $20,000. Such fully enclosed atmospheres are uncommon for light-sheet microscopes because multiple objectives contact the specimen, precluding a sealed enclosure, with the notable exception of a purpose-built enclosure reported previously[23]. In the open-source implementation of LLSM, as well as the early LLSM variant commercialized by 3i, environmental control was achieved by enclosing both the specimen and the objectives in temperature-regulated blocks through which heated or chilled water was circulated, while pH stabilization was maintained using 10 mM HEPES buffer. However, humidity and CO_2 were not controlled in this configuration. We adopt a similar strategy in Altair-LSFM, using temperature-controlled components and HEPES-buffered media to maintain a stable imaging environment. Given our ~50 mL chamber volume, evaporation is minimal even during extended imaging sessions. In contrast, OPMs, and the more recent LLSM variant commercialized by ZEISS, are inherently compatible with commercially available environmental chambers and therefore avoid many of the practical constraints associated with multi-objective light-sheet systems.

Supplementary Note 3. Light-Sheet Illumination and Acquisition Modes

LLSM Illumination Modes

The original implementation of Lattice Light-Sheet Microscopy (LLSM), introduced by Chen, Legant, Wang et al[32], is a highly flexible platform capable of generating multiple illumination patterns, each optimized for different biological specimens. This flexibility arises from its use of a spatial light modulator (SLM) to define the illumination pattern at the back focal plane of the illumination objective. The system can operate in a dithered, or time-averaged square and hexagonal lattice modes, as well as in a structured illumination mode, providing a wide range of trade-offs between resolution, field of view, and imaging speed. However, the square lattice rapidly became the most widely used due to its ease of operation and compatibility with deconvolution workflows. Indeed, in the original 2014 publication, 16 of the 20 figure subpanels utilized the square lattice configuration, while only one panel was dedicated to each of the hexagonal or structured illumination modes.

The square lattice provides strong axial confinement with minimal side-lobe energy, producing uniform, high-contrast optical sectioning that is suitable for most cellular imaging applications. The hexagonal lattice, by contrast, improves axial resolution but is accompanied by side-lobes that increase in intensity with laser propagation distance, limiting its use to particularly thin specimens. These side lobes also necessitate computational post-processing for their removal, which becomes increasingly challenging when their intensity approaches ~50% of the main lobe, a condition that can occur even for modest beam propagation lengths[10]. These beams can also operate in a structured illumination (SIM) mode, wherein the lattice pattern is stepped or phase-shifted laterally to enhance optical resolution in both the X and Z dimensions. Because the orientation of the illumination pattern is fixed, resolution along the Y axis (e.g., the laser propagation direction) remains diffraction limited. Importantly, this mode requires 5 phase-shifted images per optical section to reconstruct a single high-resolution plane, substantially increasing both illumination dose and acquisition time. The computational reconstruction is also considerably more intensive than for standard lattice modes. Although structured illumination is supported in the commercial

bioRxiv preprint doi: https://doi.org/10.1101/2025.04.08.647739; this version posted November 7, 2025. The copyright holder for this preprint (which was not certified by peer review) is the author/funder, who has granted bioRxiv a license to display the preprint in perpetuity. It is made available under aCC-BY 4.0 International license.

implementation of LLSM offered by 3i, its use in practice remains limited due to these experimental and computational demands.

The recently developed ZEISS Lattice Lightsheet 7 represents a streamlined and user-friendly evolution of the original LLSM design, prioritizing ease of operation over full optical flexibility. Unlike the earlier implementations, which supports multiple lattice geometries and structured illumination modes, the ZEISS system offers three predefined "Sinc" beam configurations. Each configuration corresponds to a specific trade-off between light-sheet length and thickness (e.g., 15 × 550 nm, 30 × 700 nm, and 100 × 1400 nm, each available with or without side lobes).

Volume Acquisition Modes

To acquire volumetric data, both the original and 3i systems can operate in sample-scanning and light-sheet-scanning modes, whereas both Altair-LSFM and the ZEISS implementation of LLSM function exclusively in a sample-scanning format. In sample scanning, the specimen is translated through a stationary light-sheet, whereas in light-sheet scanning, the light-sheet is scanned in the Z-direction through a stationary specimen synchronously with the position of the detection objective (see **Supplementary Figure 1**). Each mode offers distinct advantages and trade-offs. Sample scanning provides the simplest architecture to assemble and operate but requires shearing of the data to place the data in its proper spatial context. It is particularly advantageous for specimens with a large aspect ratio, such as highly adherent cells. In the light-sheet-scanning configuration, the sheet is scanned using a linear galvanometer conjugate to the back pupil of the illumination objective. This design necessarily introduces an additional optical fold into the illumination train, and the plane conjugate to the galvo must typically be relayed to the back pupil of the illumination objective—often with an f-theta lens and tube lens pair. The galvo sweep must also be calibrated to ensure precise synchronization between the light-sheet position and the detection objective's focal plane. Consequently, compared to sample-scanning, light-sheet-scanning increases both alignment complexity and cost. Unlike sample scanning, the acquired data are immediately in their correct spatial context, eliminating the need for post-processing shearing. Light-sheet scanning is particularly useful for specimens with a more spherical aspect ratio. In the original LLSM publication, this mode was used to image biological processes such as microtubule and histone dynamics during mitosis, *Tetrahymena thermophila* swimming behavior, T-cell immunological-synapse formation, and *C. elegans* embryogenesis. It is also advantageous for vibration-sensitive specimens, such as neutrophil-like cells migrating within a compliant extracellular matrix, where sample translation could induce unwanted mechanical perturbations in the extracellular matrix[43].

Resolution.

While the original LLSM platform supports square, hexagonal, and structured illumination modes, our discussion on resolution here remains focused on the square lattice. Importantly, for both square-lattice light sheets and Gaussian light sheets, axial resolution is tightly coupled to the propagation length of the illumination beam: Both square lattice light-sheets, which do not behave as propagation-invariant beams, and Gaussian beams broaden in diameter as a function of propagation length. Consequently, like-for-like comparisons must match detection NA, sheet thickness at focus, light sheet propagation length, and post-processing.

The original LLSM work described lattice light-sheets by inner/outer NA rather than by sheet thickness and propagation length, which complicates direct comparisons to Gaussian sheets. More recent reports with bead-based measurements (FWHM of 100-nm fluorophore beads) yielded a raw lateral and axial resolution of 312 nm × 666 nm for an excitation wavelength of 488 nm. For comparison, the original publication reported a resolution of ~230 × 370 nm, which is consistent with the use of shorter propagation length illumination beam and deconvolution. For comparison, the ZEISS implementation reports raw resolutions of 330 x 500-1000 nm and deconvolved resolutions of 290 x 450 – 900 nm, depending on the selected light-sheet configuration. The reduced resolution likely reflects the compromises inherent to imaging in an open-top geometry, which requires the use of a meniscus lens to accommodate the optical path. For Gaussian beams, a frequent source of confusion in the literature is the use of paraxial Gaussian-beam formulae to estimate thickness and propagation length at NAs where diffraction effects are non-negligible. At the illumination NAs used in Altair-LSFM, scalar/vector diffraction modeling better predicts the realized beam properties than simple Gaussian optics. Thus, when propagation length and focus thickness are matched, a diffraction-limited Gaussian sheet can deliver performance comparable to a square lattice, without introducing side lobes.

Quantitative comparisons of resolution depend not only on the optics but also on the exact algorithmic implementation of deskewing (shearing) and deconvolution. For deconvolution, outcomes are sensitive to PSF

bioRxiv preprint doi: https://doi.org/10.1101/2025.04.08.647739; this version posted November 7, 2025. The copyright holder for this preprint (which was not certified by peer review) is the author/funder, who has granted bioRxiv a license to display the preprint in perpetuity. It is made available under aCC-BY 4.0 International license.

quality, background estimation, regularization, and iteration count; moreover, most algorithms assume a non-aberrated, shift-invariant PSF, an assumption that degrades with specimen heterogeneity. In our measurements, Altair-LSFM achieves ~235 nm × 350 nm after deconvolution, comparable to the deconvolved values reported for square-lattice LLSM under matched detection NA and sampling. These reported measurements were performed on 100 nm fluorescent beads and likely reflect illumination beams optimized for the thinnest biological specimens. Thicker specimens would require longer propagation lengths and correspondingly thicker light sheets, yielding resolutions closer to those previously reported—approximately 312 × 666 nm before deconvolution or 230 × 460 nm after deconvolution, assuming a ~30% reduction in the point spread function dimensions.

Comparison with other Light-Sheet Modalities.

diSPIM attains near-isotropic resolution by imaging the same specimen from two orthogonal detection paths and fusing the volumes with multiview deconvolution. Reported performance ranges from ~330 nm isotropic in the original implementation[22] to ~380 nm isotropic in commercial systems. For cleared-tissue variants of diSPIM using mixed-immersion 17.9×/0.4-NA objectives, single-view resolutions of ~840 nm (lateral) and 4600 nm (axial) have been improved to ~800 nm isotropic after registration and one iteration of deconvolution[25]. In practice, diSPIM excels when large fields of view and improved axial sectioning are required and when multiview registration and deconvolution are acceptable parts of the workflow. High-resolution ASLM systems use aberration-free remote focusing to translate the light sheet along its propagation direction in synchrony with a camera's rolling shutter, effectively decoupling axial sectioning from field of view. Early implementations with ~0.8-NA detection achieved ~400 nm isotropic resolution[12]; adaptations for high-RI cleared samples have reported ~330 nm isotropic resolution[24]. Large field of view variants such as the mesoSPIM[44] use electro-tunable lenses to scan the illumination beam and report typical resolutions of ~2.5 µm (lateral) and ~5 µm (axial). Both diSPIM and ASLM excel when one needs to maximize the field of view while maintaining a high axial resolution.

Recent advances in OPM[45] have enabled sub-cellular imaging while preserving a single-objective, inverted geometry that is compatible with standard sample preparation, environmental chambers, and autofocus systems. A high-NA OPM has reported 299 × 731 nm (raw) and ~209 × 523 nm (after deconvolution), albeit with short working distance[13]. Longer-working-distance, water-dipping OPMs extend penetration and support optical tiling (e.g., volumes on the order of 800 × 500 × 200 µm) with raw resolutions around ~400 × 1220 nm[46]. OPM also allows novel illumination approaches, such as DaXi, which used an image flipper to perform multiview imaging with ~450 nm lateral and ~2 µm axial resolution throughout a ~3000 × 800 × 300 µm volume. Likewise, by incorporating an image rotator into the optical path, structured illumination could be leveraged to improve the lateral resolution to ~140 nm[47]. And lastly, because of the shared optical train, a single deformable mirror is capable simultaneously correcting aberrations in both the illumination and detection[48]. Given the rapid evolution of OPMs in particular, and LSFM more broadly, the comparisons provided here are intended to situate Altair-LSFM in context, not to offer an exhaustive review.

Supplementary Note 4. Data Storage and Handling

Storage costs are approximate and are expected to vary widely between institutions, depending on negotiated contracts, available infrastructure, and funding models. The cost estimates provided here are intended solely as a practical guide for planning purposes and should not be considered definitive or universally applicable. For example, large cloud providers list prices that vary from roughly $0.023 to $0.00099 per GB per month, with additional monitoring, retrieval, and transfer fees that vary by storage class. In contrast, the local computing infrastructure at UT Southwestern Medical Center charges a one-time fee of $300 per TB, which is amortized over five years. Advantageously, to reduce costs to end-users, our local file system includes an automated 100 PB tape archive: data inactive for more than 12 months are transparently migrated while remaining visible as placeholders and are automatically recalled when accessed. Our lab operates with a 130 TB quota, which costs ~$8,000 per year. In general, we typically budget $2k per year per experimentalist for storage and compute, rising to $10–12k per year for users that rely on high-end GPUs. These expenses are routinely incorporated into federally funded grant budgets. To operate within the confines of such a quota, we recommend several simple rules to manage data overhead.

- Save data in a Zarr format, which is supported by *navigate* and uses lossless blosclz compression by default.
- Immediately assess data for quality. *Navigate* automatically saves maximum-intensity projections (MIPs) that can be reviewed quickly in Fiji/ImageJ on standard computers, enabling rapid assessment of data quality. Evaluating data in two dimensions is substantially faster than loading full 3D stacks; datasets of insufficient quality should be deleted immediately to prevent unnecessary storage costs.

bioRxiv preprint doi: https://doi.org/10.1101/2025.04.08.647739; this version posted November 7, 2025. The copyright holder for this preprint (which was not certified by peer review) is the author/funder, who has granted bioRxiv a license to display the preprint in perpetuity. It is made available under aCC-BY 4.0 International license.

- Store only the raw image data. Saving Postprocessing routines, such as deconvolution, increase the storage footprint. Instead, we reprocess data on demand using high-performance tools such as PetaKit5D[31]. Importantly, to support reproducibility, one must record processing parameters such as deconvolution iterations and point-spread functions alongside each dataset to enable faithful reprocessing.

Supplementary Note 5. Comparison of *navigate* with other Open-Source Microscope Control Software.

A wide variety of open-source software packages are available for microscope control, the most popular of which is Micro-Manager[49], a highly extensible and widely adopted platform. Both *navigate* and Micro-Manager provide user-friendly graphical interfaces and support image-based feedback control, making them accessible to a broad community of users. Micro-Manager's greatest strength lies in its extensive library of device drivers, which supports a vast array of commercial hardware from multiple manufacturers. Although Micro-Manager has been successfully used to control light-sheet microscopes, it typically requires manual hardware configuration and custom scripting to implement advanced or non-standard acquisition workflows.

In contrast, *navigate* was developed from the ground up to provide turnkey support for multiple light-sheet architectures, including both sample-scanning and light-sheet–scanning LSFM configurations, as well as ASLM and OPM. While its hardware library is more focused than Micro-Manager's, *navigate* was developed in tandem with the Altair-LSFM hardware, enabling tightly integrated, preconfigured acquisition routines optimized for this system. This co-design minimizes setup time, eliminates the need for user-side customization, and ensures reliable synchronization across devices. The result is a software environment that provides robust performance, efficient data handling, and streamlined operation for non-expert users.

Navigate also offers advanced data management capabilities through native support for multiple file formats, including TIFF, HDF5, N5, and Zarr. Each file is saved with embedded Open Microscopy Environment (OME) metadata and is fully compatible with BigDataViewer, facilitating interoperability with downstream image analysis pipelines. Additionally, *navigate's* modular Python-based architecture allows expert users to extend functionality, integrate new devices, and leverage the extensive Python ecosystem for intelligent or adaptive acquisition workflows.

Although *navigate* is provided as the default control platform for Altair-LSFM, the system can, in principle, be configured to operate with Micro-Manager or other open-source control frameworks. We welcome and encourage community-led efforts to build such compatibility. Ultimately, our goal with *navigate* is to provide a ready-to-use, flexible, and extensible control environment that lowers the barrier to advanced light-sheet imaging while supporting continued community innovation and customization.

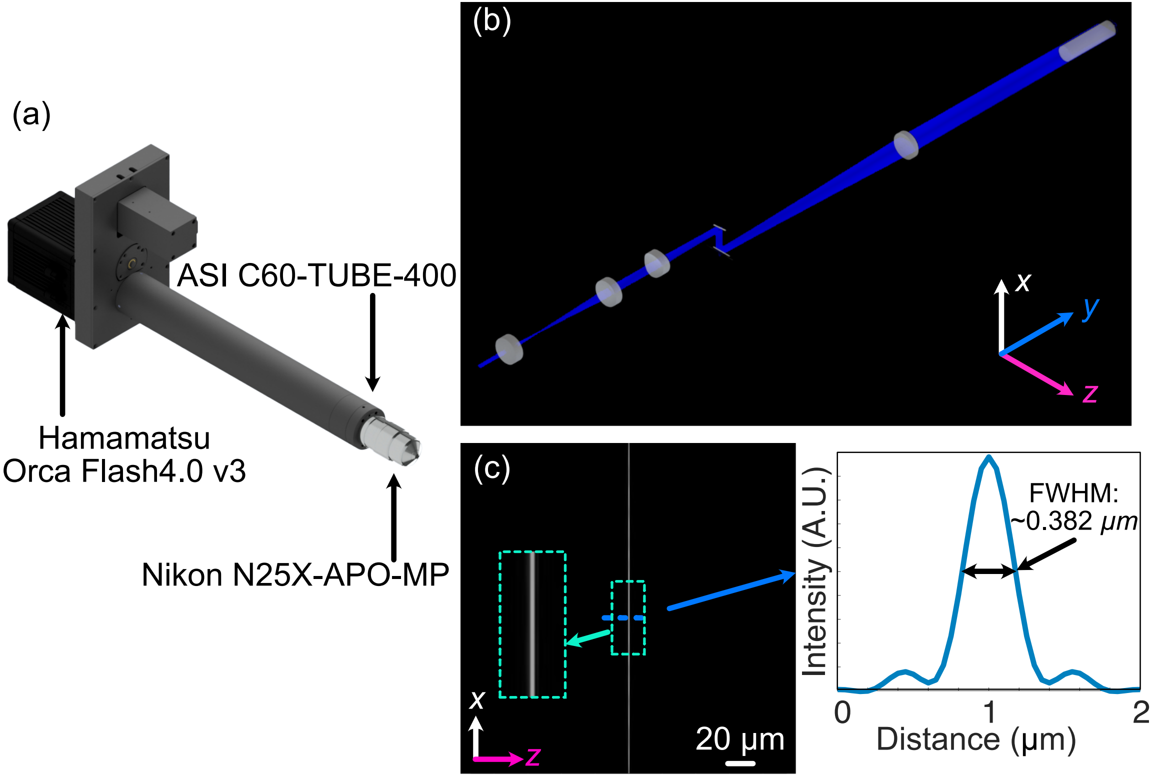
(a)
ASI C60-TUBE-400
Hamamatsu
Orca Flash4.0 v3
Nikon N25X-APO-MP
(b)
x
y
z
(c)
x
z
20 µm
FWHM:
~0.382 µm
Intensity (A.U.)
0
1
2
Distance (µm)

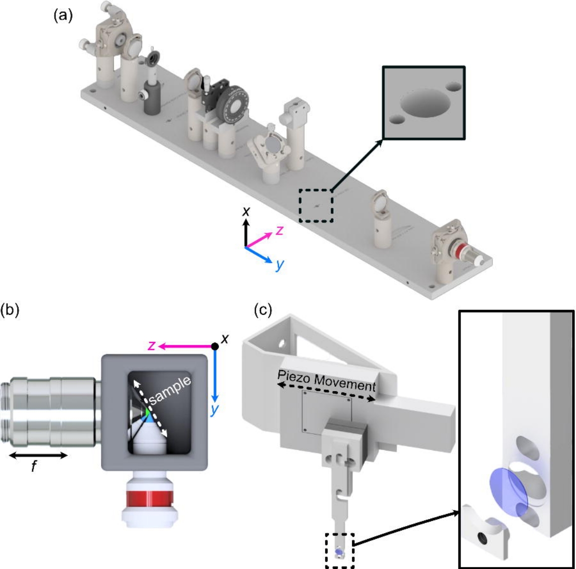

(a)
x
z
y
(b)
z
x
y
sample
f
(c)
Piezo Movement

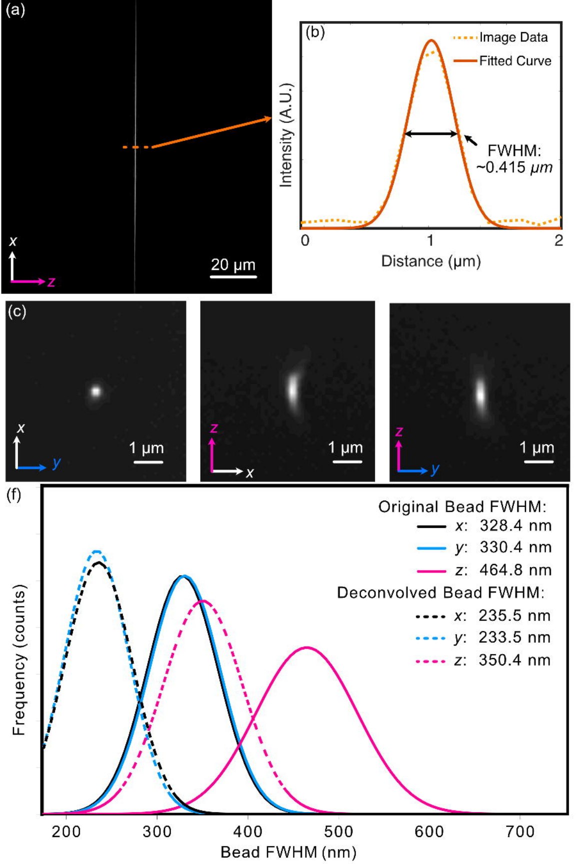
(a)
x
z
20 µm
(b)
Image Data
Fitted Curve
Intensity (A.U.)
FWHM:
~0.415 µm
0
1
2
Distance (µm)
(c)
x
y
1 µm
z
x
1 µm
z
y
1 µm
(f)
Original Bead FWHM:
x: 328.4 nm
y: 330.4 nm
z: 464.8 nm
Deconvolved Bead FWHM:
x: 235.5 nm
y: 233.5 nm
z: 350.4 nm
Frequency (counts)
200
300
400
500
600
700
Bead FWHM (nm)

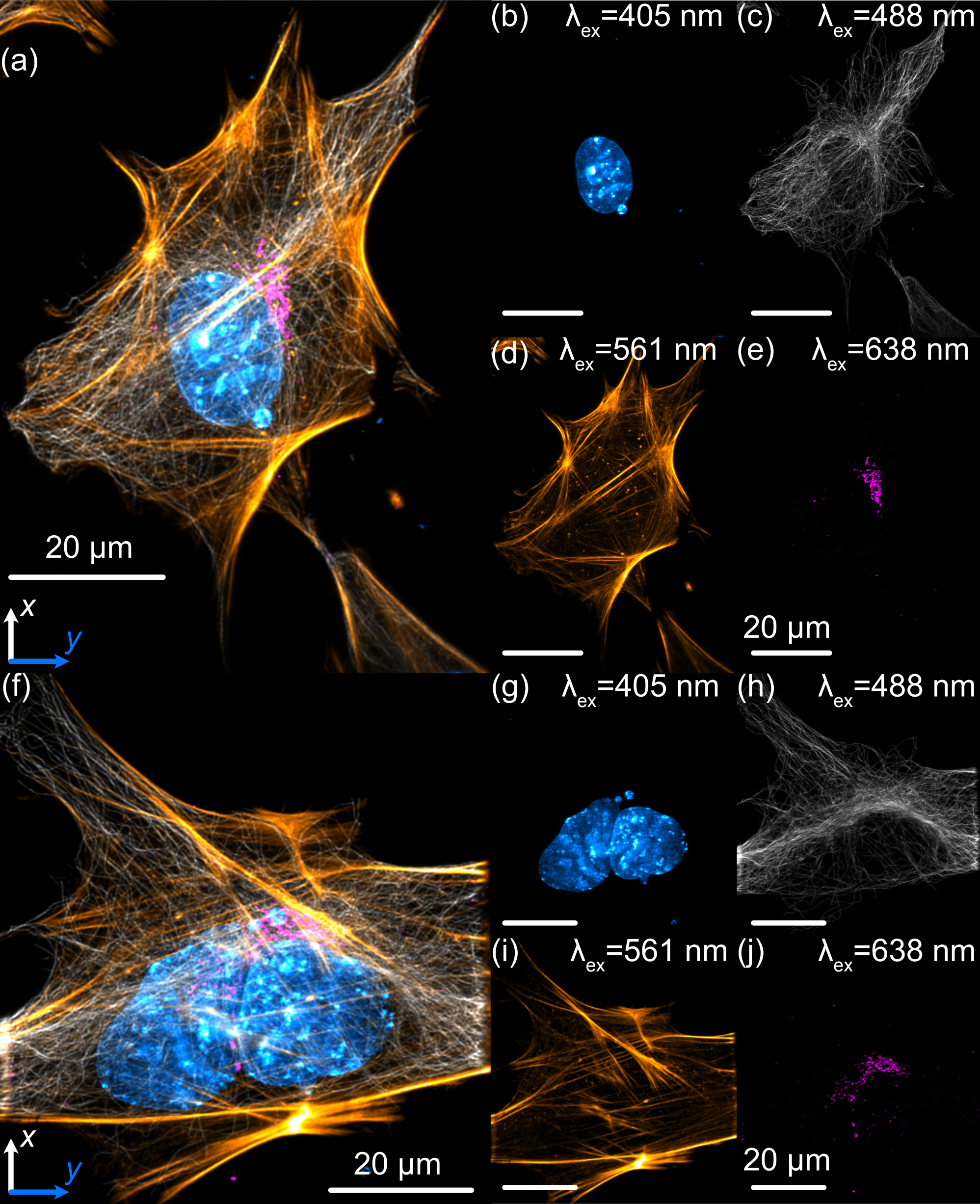

(a)
20 μm
x
y
(b) λex=405 nm
(c) λex=488 nm
(d) λex=561 nm
(e) λex=638 nm
20 μm
(f)
x
y
20 μm
(g) λex=405 nm
(h) λex=488 nm
(i) λex=561 nm
(j) λex=638 nm
20 μm

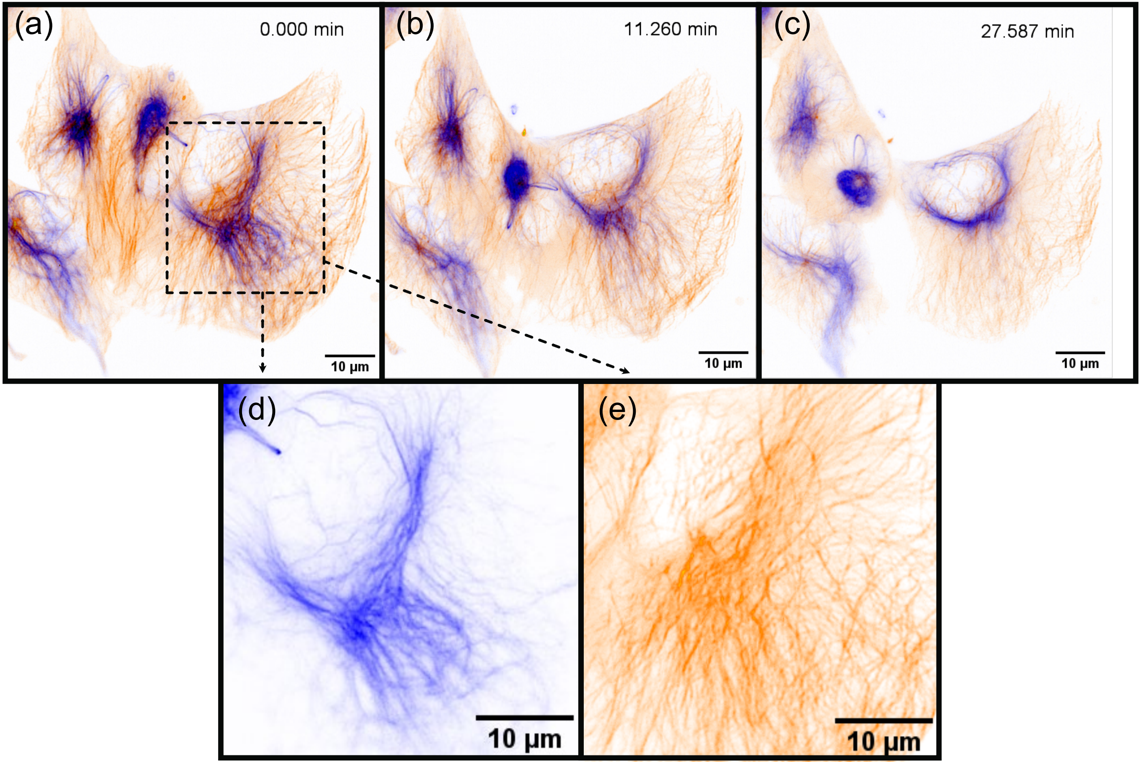
(a)
0.000 min
10 µm
(b)
11.260 min
10 µm
(c)
27.587 min
10 µm
(d)
10 µm
(e)
10 µm

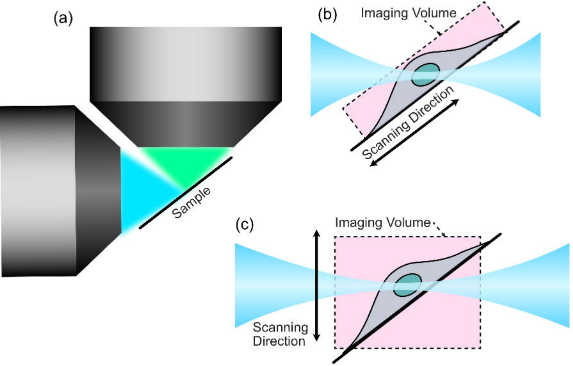
(a)
Sample
(b)
Imaging Volume
Scanning Direction
(c)
Imaging Volume
Scanning
Direction

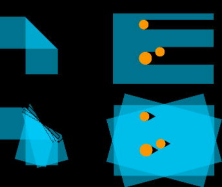

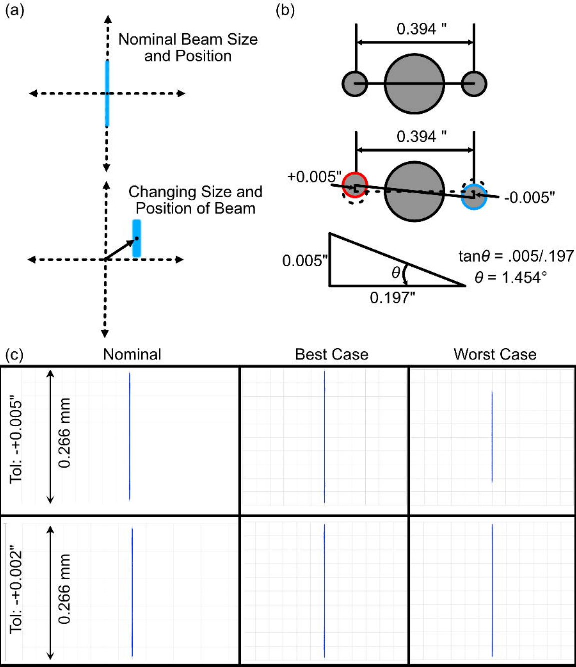

(a)
Nominal Beam Size and Position
Changing Size and Position of Beam
(b)
0.394 "
0.394 "
+0.005"
-0.005"
0.005"
θ
0.197"
tanθ = .005/.197
θ = 1.454°
(c)
Nominal
Best Case
Worst Case
Tol: -+0.005"
0.266 mm
Tol: -+0.002"
0.266 mm

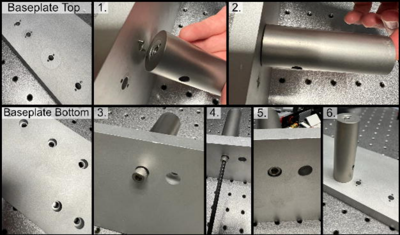
Baseplate Top
1.
2.
Baseplate Bottom
3.
4.
5.
6.

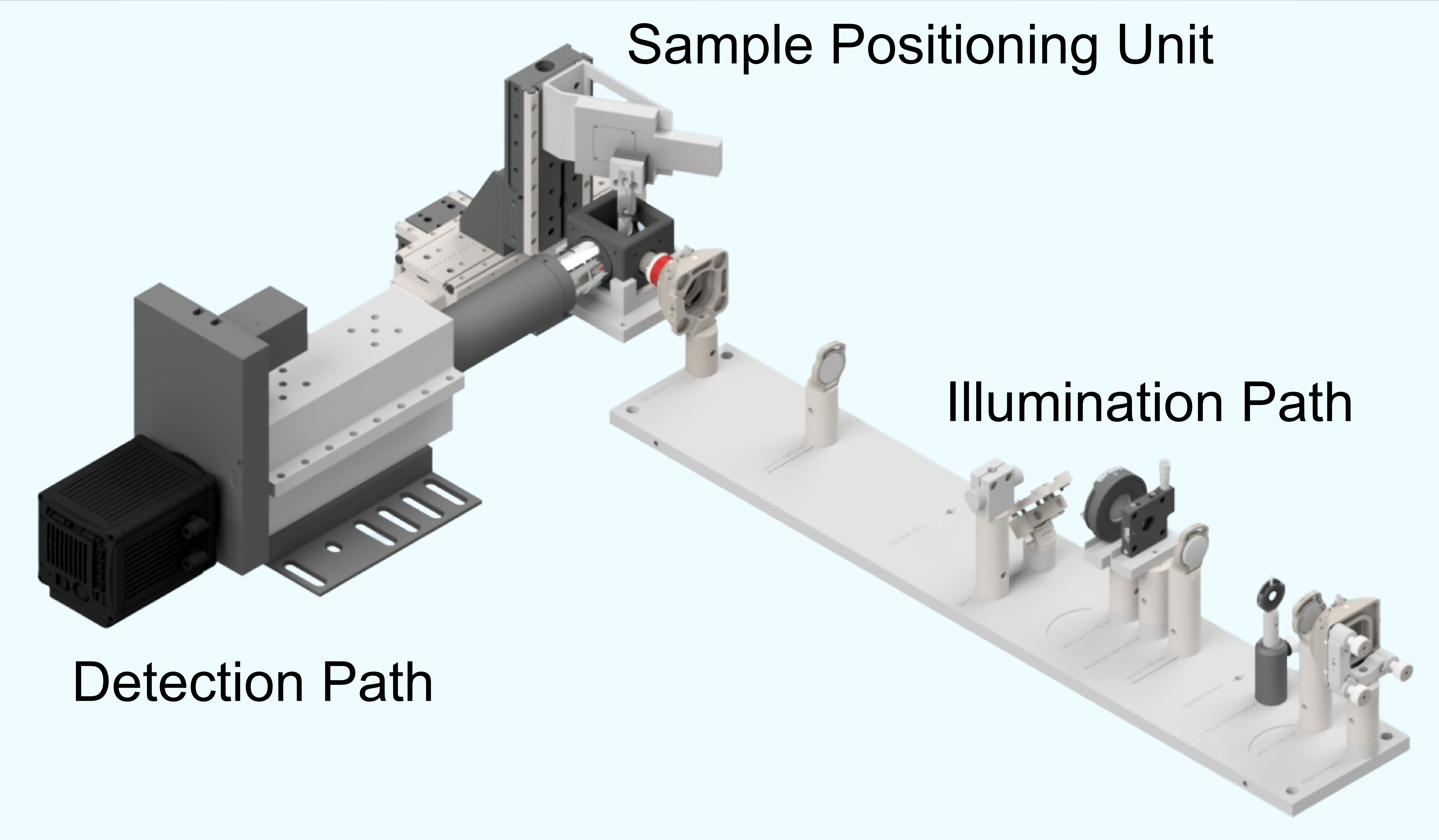
Sample Positioning Unit
Illumination Path
Detection Path

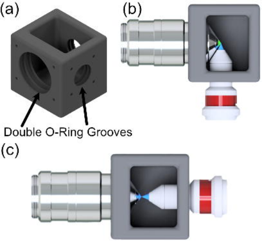
(a)
(b)
Double O-Ring Grooves
(c)

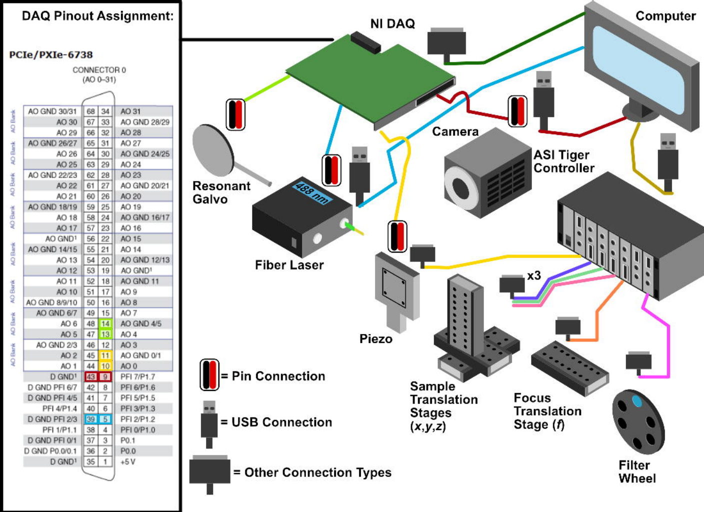

DAQ Pinout Assignment:
PCIe/PXIe-6738
CONNECTOR 0
(AO 0–31)
AO GND 30/31 68 34 AO 31
AO 30 67 33 AO GND 28/29
AO 29 66 32 AO 28
AO GND 26/27 65 31 AO 27
AO 26 64 30 AO GND 24/25
AO 25 63 29 AO 24
AO GND 22/23 62 28 AO 23
AO 22 61 27 AO GND 20/21
AO 21 60 26 AO 20
AO GND 18/19 59 25 AO 19
AO 18 58 24 AO GND 16/17
AO 17 57 23 AO 16
AO GND1 56 22 AO 15
AO GND 14/15 55 21 AO 14
AO 13 54 20 AO GND 12/13
AO 12 53 19 AO GND1
AO 11 52 18 AO GND 11
AO 10 51 17 AO 9
AO GND 8/9/10 50 16 AO 8
AO GND 6/7 49 15 AO 7
AO 6 48 14 AO GND 4/5
AO 5 47 13 AO 4
AO GND 2/3 46 12 AO 3
AO 2 45 11 AO GND 0/1
AO 1 44 10 AO 0
D GND1 43 9 PFI 7/P1.7
D GND PFI 6/7 42 8 PFI 6/P1.6
D GND PFI 4/5 41 7 PFI 5/P1.5
PFI 4/P1.4 40 6 PFI 3/P1.3
D GND PFI 2/3 39 5 PFI 2/P1.2
PFI 1/P1.1 38 4 PFI 0/P1.0
D GND PFI 0/1 37 3 P0.1
D GND P0.0/0.1 36 2 P0.0
D GND1 35 1 +5 V
AO Bank
NI DAQ
Computer
Resonant Galvo
488 nm
Fiber Laser
Camera
ASI Tiger Controller
Piezo
x3
Sample Translation Stages (x,y,z)
Focus Translation Stage (f)
Filter Wheel
= Pin Connection
= USB Connection
= Other Connection Types

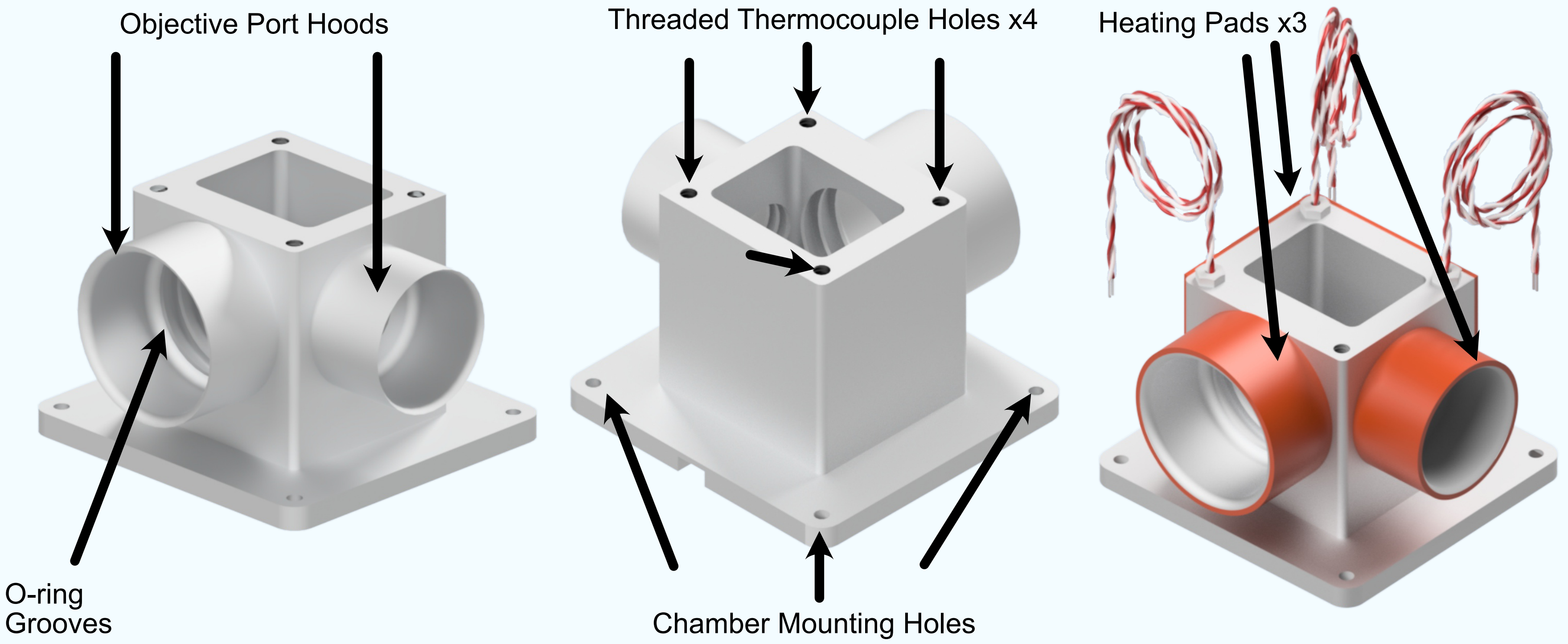
Objective Port Hoods
O-ring
Grooves
Threaded Thermocouple Holes x4
Chamber Mounting Holes
Heating Pads x3

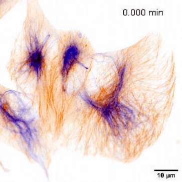
0.000 min
10 µm
